# BDSF Analogues Inhibit Quorum Sensing-Regulated Biofilm Production in *Xylella fastidiosa*

**DOI:** 10.3390/microorganisms12122496

**Published:** 2024-12-04

**Authors:** Conor Horgan, Clelia Baccari, Michelle O’Driscoll, Steven E. Lindow, Timothy P. O’Sullivan

**Affiliations:** 1 School of Chemistry, University College Cork, T12 YN60 Cork, Ireland; conor.horgan@umail.ucc.ie (C.H.); 121128531@umail.ucc.ie (M.O.); 2 School of Pharmacy, University College Cork, T12 YN60 Cork, Ireland; 3 Analytical and Biological Chemistry Research Facility, University College Cork, T12 YN60 Cork, Ireland; 4 Department of Plant and Microbial Biology, University of California, Berkeley, CA 94720, USA; clelia.baccari@berkeley.edu

**Keywords:** *Xylella fastidiosa*, diffusible signal factor, quorum sensing, biofilms, bioisosteres

## Abstract

*Xylella fastidiosa* is an aerobic, Gram-negative bacterium that is responsible for many plant diseases. The bacterium is the causal agent of Pierce’s disease in grapes and is also responsible for citrus variegated chlorosis, peach phony disease, olive quick decline syndrome and leaf scorches of various species. The production of biofilm is intrinsically linked with persistence and transmission in *X. fastidiosa*. Biofilm formation is regulated by members of the Diffusible Signal Factor (DSF) quorum sensing signalling family which are comprised of a series of long chain *cis*-unsaturated fatty acids. This article describes the evaluation of a library of *N*-acyl sulfonamide bioisosteric analogues of BDSF, *Xf*DSF1 and *Xf*DSF2 for their ability to control biofilm production in *X. fastidiosa*. The compounds were screened against both the wild-type strain Temecula and an rpfF* mutant which can perceive but not produce *Xf*DSF. Planktonic cell abundance was measured via OD600 while standard crystal violet assays were used to determine biofilm biomass. Several compounds were found to be effective biofilm inhibitors depending on the nature of the sulfonamide substituent. The findings reported here may provide future opportunities for biocontrol of this important plant pathogen.

## 1. Introduction

*Xylella fastidiosa* is an aerobic, Gram-negative bacterium that is responsible for many plant diseases. According to the European Commission, *X. fastidiosa* “is one of the most dangerous plant bacteria worldwide” [1]. It was first reported by Wells et al. as the causal agent of Pierce’s disease in grapes [2]. The bacterium is also responsible for citrus variegated chlorosis, peach phony disease, olive quick decline syndrome (OQDS) and leaf scorches of almond, Japanese plum, elm, sycamore, oak, mulberry and maple. Common symptoms exhibited by plants infected by *X. fastidiosa* include scorching and wilting of foliage and stunted growth in leaves, fruit and plant height, followed by the eventual death of the plant.

**Figure 1:**
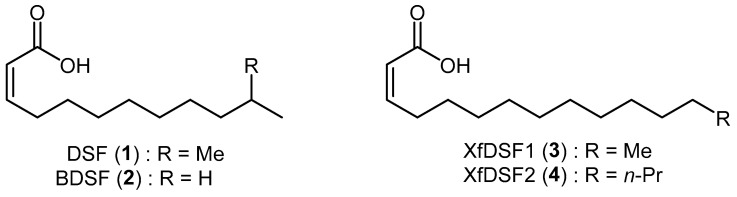
Structures of DSF/BDSF and *Xf*DSF1/2 messenger molecules.

Biofilm formation in *X. fastidiosa* is a group behaviour regulated by members of the Diffusible Signal Factor family of signalling molecules. These messenger molecules are comprised of *cis*-unsaturated fatty acids of which *cis*-11-methyl-2-dodecenoic acid (**1**), known as Diffusible Signal Factor or DSF, and *cis*-2-dodecenoic acid (**2**), known as *Burkholderia* Diffusible Signal Factor or BDSF, are commonly employed by a wide range of bacteria for the regulation of diverse biological functions including biofilm formation, antibiotic tolerance and virulence (Figure 1) [3,4]. Although *X. fastidiosa* in principle is capable of producing many long chain saturated and unsaturated fatty acids, only two biologically active DSF derivatives have been isolated from the bacterium, namely 14-carbon *Xf*DSF1 (**3**) and 16-carbon *Xf*DSF2 (**4**), where *Xf* refers to *Xylella fastidiosa* [5,6]. The biosynthesis and perception of *Xf*DSF signalling molecules in *X. fastidiosa* is very similar to that of DSF in *X. campestris*. RpfF is the key protein involved in the biosynthetic pathway, using 3-hydroxyacyl-acyl carrier protein dehydratase activity to introduce the *cis*-unsaturated double bond at the α,β-position. Additionally, thioesterase activity hydrolyses the thioester bond to release the carboxylic acid [7]. RpfC is the main sensor protein. Experiments have shown that, unlike *X. campestris*, RpfF also plays a role in signal perception in *X. fastidiosa* but this is probably a secondary pathway [8]. Signal detection activates cyclic di-GMP phosphodiesterase activity in RpfG which, in turn, triggers the expression of target genes such as hxfA and hxfB which are responsible for cell–cell aggregation and biofilm formation [9,10]. Interestingly, the introduction of *rpfF* mutations has an opposite effect on virulence in *X*. *fastidiosa* versus *X*. *campestris* [11]. *rpfF* mutants, which were incapable of DSF production and subsequently biofilm formation, caused early onset, severe scorching/wilting symptomatic of hypervirulence compared to the WT strain. The enhanced virulence of mutants incapable of DSF-mediated gene regulation was associated with reduced adhesiveness of the cells to surfaces, thus facilitating movement between xylem vessels to more extensively colonise infected plants. By contrast, in *X*. *campestris*, the absence of DSF by way of *rpfF* mutation results in hypovirulence. It is, therefore, reasonable to suggest that *X*. *fastidiosa* uses quorum sensing to partition its populations into plants on the basis of local DSF concentrations to enable to coexistence of hyperadhesive cells capable of acquisition and transmission by insect vectors as well as cells of low adhesiveness that are capable of rapid spread throughout the plant [11].

While *X. campestris* is most responsive to DSF molecules with chains between 10 and 14 carbons [3], *X. fastidiosa*, by contrast, responds to molecules with chain lengths of 12 to 18 carbons [6]. *X. fastidiosa* also responds to BDSF, albeit at concentrations 3 and 20 times higher than the native signals *Xf*DSF1 and *Xf*DSF2, respectively. Of the two biologically active DSF species produced by *X. fastidiosa*, 16-carbon *Xf*DSF2 is more active as a signalling molecule. *X. fastidiosa* responds to *Xf*DSF2 at concentrations six times lower than *Xf*DSF1. Prior research also demonstrated that when an *X. fastidiosa rpfF* deletion mutant was exposed to *Xf*DSF2, expression of the *hxfB* gene was approximately 30-fold higher when compared to exposure to *Xf*DSF1. Both *Xf*DSF1 and *Xf*DSF2 stimulated biofilm formation and increased the attachment of cells to a liquid–air interface of shaken cultures in glass tubes. After 24 h of incubation, the number of cells attached to the glass was 2.8- or 1.7-fold higher in the presence of *Xf*DSF2 or *Xf*DSF1, respectively, than in the control, indicating that *Xf*DSF2 more effectively induces adhesion. This may account in part for the association between increased exposure to *Xf*DSF and increased transmission.

There are limited examples in the literature of successful inhibition of biofilm formation in *X. fastidiosa*. Given that DSF-derived molecules play a major role in controlling biofilm formation in this bacterium*,* which, in turn, affects the adhesiveness of cells that is central to both virulence and transmission, we wondered if synthetic analogues of DSF might have an inhibitory effect. We have previously established that *N*-acyl sulfonamide derivatives of BDSF can successfully interfere with biofilm formation and quorum sensing in a variety of DSF-sensitive bacteria such as *P. aeruginosa*, *B. cepacia* and *A. baumannii* [12,13,14]. In this article, we describe the evaluation of these BDSF bioisosteres for their ability to inhibit *X. fastidiosa* biofilm production for the first time. In addition, this paper also describes the preparation of a series of novel 14- and 16-carbons analogues of *Xf*DSF1 and *Xf*DSF2 to elucidate the importance of the carbon chain. The inhibitory activity of these longer chain analogues is also measured, allowing for comparison with the corresponding 12-carbon BDSF derivatives.

## 2. Materials and Methods

### 2.1. Synthesis

^1^H NMR and ^13^C NMR spectra were recorded on Bruker Avance 300/400 MHz NMR spectrometers. Compounds were purified by flash chromatography using Kieselgel 60/0.040–0.063 mm silica gel. 1-Tridecyne and 1-undecyne were sourced from Fisher Scientific, Ballycoolen, Dublin, Ireland. Sulfonamides were sourced from Fluorochem Ltd., Glossop, Derbyshire, UK. Triethylamine, platinum (IV) oxide, copper iodide, bis(triphenylphosphine)palladium(II) dichloride and reaction solvents were sourced from Sigma-Aldrich, Gillingham, Dorset, UK. All commercial reagents/solvents were used without additional purification unless otherwise stated.

#### 2.1.1. Synthesis of Methyl 2-(Undec-1-yn-1-yl)benzoate (**29**)

CuI (5 mg, 0.030 mmol, 5 mol%) and bis(triphenylphosphine)palladium(II) dichloride (12 mg, 0.018 mmol, 3 mol%) were added to an oven-dried reaction tube. Distilled Et_3_N (0.8 mL, 5.703 mmol, 9.50 eq.) and **11** (156 mg, 0.595 mmol, 1.00 eq.) in degassed anhydrous MeCN (6 mL) were subsequently added before addition of 1-undecyne (0.14 mL, 0.709 mmol, 1.20 eq.). The reaction mixture was heated via microwave irradiation to 100 °C (120 W) for 1 h. The reaction mixture was filtered through a celite pad, quenched with water and extracted with diethyl ether (3 × 30 mL). The organic layers were combined and washed with water (20 mL), brine (20 mL) and dried with MgSO_4_. The mixture was then filtered and the solvent removed in vacuo. The crude residue was subjected to flash chromatography using hexane-diethyl ether (100:0–98:2) to afford **29** as a colourless oil (143 mg, 0.50 mmol, 84%).

**^1^H NMR** (400 MHz, CDCl_3_) δ 0.79 (t, 3H, *J* = 6.9 Hz, C11′), 1.11–1.29 (m, 10H, C6′–C10′), 1.33–1.44 (m, 2H, C5′), 1.54 (tt, 2H, *J* = 7.6 Hz, C4′), 2.38 (t, 2H, *J* = 7.1 Hz, C3′), 3.81 (s, 3H, C8), 7.20 (ddd, 1H, *J* = 7.6, 1.3 Hz, C5), 7.31 (ddd, 1H, *J* = 7.6, 1.4 Hz, C4), 7.41 (dd, 1H, *J* = 7.8, 1.1 Hz, C3), 7.78 (dd, 1H, *J* = 7.9, 1.3 Hz, C6).

**^13^C NMR** (100 MHz, CDCl_3_) δ 14.1 (C11′, CH_3_), 19.8 (C3′, CH_2_), 22.7 (C10′, CH_2_), 28.7 (CH_2_), 29.0 (CH_2_), 29.2 (CH_2_), 29.3 (CH_2_), 29.5 (CH_2_), 31.9 (C9′, CH_2_), 52.0 (C8, CH_3_), 79.2 (C1′), 96.0 (C2′), 124.5 (C2), 127.1 (C5, CH), 130.1 (C6, CH), 131.4 (C4, CH), 131.9 (C1), 134.2 (C3, CH), 166.9 (C7).

**IR** (ATR) $\bar{\upsilon }$*_max_* cm^−1^ 2925, 2854, 2226, 1735, 1718, 1484, 1447, 1292, 1249, 1128, 1083, 966, 756, 701.

**HRMS** (ESI) *m*/*z*: [M+H] Calcd for C_19_H_26_O_2_ 287.2005; Found 287.2001.

#### 2.1.2. Synthesis of Methyl 2-Undecylbenzoate (**30**)

**29** (143 mg, 0.500 mmol, 1.00 eq.) in MeOH (4 mL) was added to an oven-dried RBF containing PtO_2_.H_2_O (6 mg, 0.025 mmol, 5 mol%). The RBF was next evacuated and then backfilled with H_2_. The reaction mixture was stirred for 1 h under a H_2_ atmosphere. Following filtration through a celite pad, the solvent was removed in vacuo to afford **30** as a yellow oil (139 mg, 0.479 mmol, 96%).

**^1^H NMR** (400 MHz, CDCl_3_) δ 0.88 (t, 3H, *J* = 6.6 Hz, C11′), 1.14–1.44 (m, 16H, C3′-C10′), 1.49–1.67 (m, 2H, C2′), 2.93 (t, 2H, *J =* 7.8 Hz, C1′), 3.88 (s, 3H, C8), 7.15–7.29 (m, 2H, C3, C5), 7.40 (ddd, 1H, *J* = 1.3, 7.5 Hz, C4), 7.84 (dd, 1H, *J* = 1.1, 7.8 Hz, C6).

**^13^C NMR** (100 MHz, CDCl_3_) δ 14.1 (C11′, CH_3_), 22.7 (C10′, CH_2_), 29.4 (CH_2_), 29.5 (CH_2_), 29.6 (CH_2_), 29.7 (CH_2_), 29.7 (CH_2_), 29.8 (CH_2_), 31.8 (C2′, CH_2_), 31.9 (C9′, CH_2_), 34.5 (C1′, CH_2_), 51.8 (C8, CH_3_), 125.6 (C5, CH), 129.5 (C1), 130.5 (C6, CH), 130.9 (C3, CH), 131.8 (C4, CH), 144.7 (C2), 168.3 (C7).

**IR** (ATR) $\bar{\upsilon }$*_max_* cm^−1^ 2924, 2854, 1726, 1601, 1575, 1464, 1433, 1256, 1189, 1104, 1081, 750, 710.

**HRMS** (ESI) *m*/*z*: [M+H] Calcd for C_19_H_30_O_2_ 291.2318; Found 291.2313.

#### 2.1.3. Synthesis of 2-Undecylbenzoic Acid (**31**)

LiOH (115 mg, 4.792 mmol, 10.00 eq.) was added to an RBF containing methyl 2-undecylbenzoate (**30**) (139 mg, 0.479 mmol, 1.00 eq.) in THF/MeOH/water 3:1:1 (15 mL). The reaction mixture was heated to reflux for 16 h and then allowed to cool to r.t. before the solvent was removed in vacuo. Following addition of water (15 mL), 2M aqueous HCl (15 mL) was added until the solution reached pH 2, before extraction with diethyl ether (5 × 30 mL). The organic layers were combined, washed with brine and dried over MgSO_4._ Following filtration, the solvent was removed in vacuo to afford **31** as a white solid (127 mg, 0.46 mmol, 96%), mp 37–39 °C.

**^1^H NMR** (300 MHz, CDCl_3_) δ 0.87 (t, 3H, *J* = 6.7 Hz, C11′), 1.16–1.48 (m, 16H, C3′–10′), 1.55–1.72 (m, 2H, C2′), 3.02 (t, 2H, *J* = 7.8 Hz, C1′), 7.20–7.33 (m, 2H, C3, C5), 7.46 (ddd, 1H, *J* = 7.5, 1.4 Hz, C4), 8.03 (dd, 1H, *J* = 8.5, 1.6 C6).

**^13^C NMR** (75 MHz, CDCl_3_) δ 14.1 (C11′, CH_3_), 22.7 (C10′, CH_2_), 29.4 (CH_2_), 29.5 (CH_2_), 29.6 (CH_2_), 29.7 (CH_2_), 29.7 (CH_2_), 29.7 (CH_2_), 31.8 (C2′, CH_2_), 31.9 (C9′, CH_2_), 34.6 (C1′, CH_2_), 125.8 (C5, CH), 128.2 (C1), 131.2 (C3, CH), 131.6 (C6, CH), 132.8 (C4, CH), 146.0 (C2), 173.5 (C7).

**IR** (ATR) $\bar{\upsilon }$*_max_* cm^−1^ 2923, 2853, 2649, 1691, 1575, 1457, 1405, 1299, 1267, 929, 748, 723, 659, 560.

**HRMS** (ESI) *m*/*z*: [M+H] Calcd for C_18_H_28_O_2_ 277.2162; Found 277.2159.

#### 2.1.4. General Procedure for the Synthesis of *N*-(Sulfonyl)-2-undecylbenzamides

2-Undecylbenzoic acid (**31**) (25 mg, 0.090 mmol, 1.00 eq.) and DMAP (12 mg, 0.099 mmol, 1.10 eq.) were dissolved in dichloromethane (5 mL). The mixture was cooled to 0 °C before addition of the chosen sulfonamide reagent (0.085 mmol, 0.95 eq.). After 15 min, DCC (20 mg, 0.099 mmol, 1.10 eq.) was added, and stirring at r.t. was continued for 16 h. The urea byproduct was removed by celite pad filtration, and the solvent was removed in vacuo. The residue was dissolved in diethyl ether (10 mL), poured onto 2M aqueous HCl (20 mL) and extracted with diethyl ether (3 × 30 mL). The organic layers were combined, washed with brine and dried over MgSO_4_. Following filtration, the solvent was removed in vacuo, and the crude residue was subjected to flash chromatography using a suitable eluent.

#### 2.1.5. *N*-(*Tert*-butylsulfonyl)-2-undecylbenzamide (**32**)

The title compound was synthesised using 2-undecylbenzoic acid (**31**) (25 mg, 0.090 mmol, 1.00 eq.), DMAP (12 mg, 0.099 mmol, 1.10 eq.), *tert*-butylsulfonamide (12 mg, 0.085 mmol, 0.95 eq.) and DCC (20 mg, 0.099 mmol, 1.10 eq.) in DCM (5 mL). The crude residue was subjected to flash chromatography using hexane-diethyl ether (70:30) to afford **32** as a white solid (24 mg, 0.060 mmol, 70%), mp 67–68 °C.

**^1^H NMR** (400 MHz, CDCl_3_) δ 0.88 (t, 3H, *J* = 6.7 Hz, C11′), 1.16–1.39 (m, 16H, C3′-C10′), 1.53–1.68 (m, 2H, overlapped, C2′), 1.56 (s, 9H, C2″–C4″), 2.80 (t, 2H, *J* = 7.9 Hz, C1′), 7.20–7.33 (m, 2H, C3, C5), 7.36–7.49 (m, 2H, C4, C6).

**^13^C NMR** (100 MHz, CDCl_3_) δ 14.1 (C11′, CH_3_), 22.7 (C10′, CH_2_), 24.6 (C2″–C4″, 3 × CH_3_), 29.3 (CH_2_), 29.5 (CH_2_), 29.6 (CH_2_), 29.6 (CH_2_), 29.6 (CH_2_), 29.6 (CH_2_), 31.9 (C2′, CH_2_), 32.0 (C9′, CH_2_), 33.4 (C1′, CH_2_), 62.5 (C1″), 126.0 (C5, CH), 126.8 (C6, CH), 130.9 (C3, CH), 131.5 (C4, CH), 133.3 (C1), 142.4 (C2), 166.6 (C7).

**IR** (ATR) $\bar{\upsilon }$*_max_* cm^−1^ 3240, 2923, 2853, 1714, 1447, 1428, 1332, 1140, 1056, 844, 749, 649, 567, 511.

**HRMS** (ESI) *m*/*z*: [M+H] Calcd for C_22_H_37_NO_3_S 396.2567; Found 396.2563.

#### 2.1.6. *N*-((4-Chlorophenyl)sulfonyl)-2-undecylbenzamide (**33**)

The title compound was synthesised using 2-undecylbenzoic acid (**31**) (25 mg, 0.090 mmol, 1.00 eq.), DMAP (12 mg, 0.099 mmol, 1.10 eq.), 4-chlorobenzenesulfonamide (16 mg, 0.085 mmol, 0.95 eq.) and DCC (20 mg, 0.099 mmol, 1.10 eq.) in DCM (5 mL). The crude residue was subjected to flash chromatography using hexane-diethyl ether (70:30) to afford **33** as a white solid (27 mg, 0.060 mmol, 70%), mp 70–72 °C.

**^1^H NMR** (400 MHz, CDCl_3_) δ 0.88 (t, 3H, *J* = 6.9 Hz, C11′), 1.08–1.38 (m, 18H, C2′-C10′), 2.62 (t, 2H, *J* = 7.9 Hz, C1′), 7.17–7.25 (m, 2H, C3, C5), 7.32–7.44 (m, 2H, C4, C6), 7.49–7.59 (m, 2H, C3″, C5″), 8.04–8.14 (m, 2H, C2″, C6″), 8.59 (s, 1H, N–H).

**^13^C NMR** (100 MHz, CDCl_3_) δ 14.2 (C11′, CH_3_), 22.7 (C10′, CH_2_), 29.3 (CH_2_), 29.4 (CH_2_), 29.5 (CH_2_), 29.6 (CH_2_), 29.7 (2 × CH_2_), 31.7 (C2′, CH_2_), 31.9 (C9′, CH_2_), 33.2 (C1′, CH_2_), 126.0 (C5, CH), 127.1 (C6, CH), 129.3 (C3″, C5″, 2 × CH), 130.1 (C2″, C6″, 2 × CH), 130.9 (C3, CH), 131.8 (C4, CH), 131.9 (C1), 136.8 (C1″), 140.8 (C4″), 142.8 (C2), 166.4 (C7).

**IR** (ATR) $\bar{\upsilon }$*_max_* cm^−1^ 3254, 2925, 2854, 1703, 1587, 1476, 197, 1333, 1162, 1089, 1014, 823, 755, 624, 537, 485.

**HRMS** (ESI) *m*/*z*: [M+H] Calcd for C_24_H_32_ClNO_3_S 450.1864; Found 450.1860.

#### 2.1.7. Synthesis of Methyl 2-(Tridec-1-yn-1-yl)benzoate (**34**)

CuI (5 mg, 0.030 mmol, 5 mol%) and bis(triphenylphosphine)palladium(II) dichloride (12 mg, 0.018 mmol, 3 mol%) were added to an oven-dried reaction tube. Distilled Et_3_N (0.8 mL, 5.703 mmol, 9.50 eq.) and **11** (156 mg, 0.595 mmol, 1.00 eq.) in degassed anhydrous MeCN (6 mL) were subsequently added before addition of 1-tridecyne (0.16 mL, 0.702 mmol, 1.20 eq.). The reaction mixture was heated via microwave irradiation at 100 °C (120 W) for 1 h. The reaction mixture was filtered through a celite pad, quenched with water and extracted with diethyl ether (3 × 30 mL). The organic layers were combined and washed with water (20 mL), brine (20 mL) and dried with MgSO_4_. The mixture was then filtered and the solvent removed in vacuo. The crude residue was subjected to flash chromatography using hexane-diethyl ether (100:0–98:2) to afford **34** as a yellow oil (150 mg, 0.478 mmol, 80%).

**^1^H NMR** (300 MHz, CDCl_3_) δ 0.88 (t, 3H, *J* = 6.8 Hz, C13′), 1.17–1.39 (m, 14H, C6′-C12′), 1.39–1.53 (m, 2H, C5′), 1.53–1.69 (m, 2H, C4′), 2.47 (t, 2H, *J* = 7.1 Hz, C3′), 3.91 (s, 3H, C8), 7.30 (ddd, 1H, *J* = 7.6, 1.4 Hz, C5), 7.41 (ddd, 1H, *J* = 7.5, 1.5 Hz, C4), 7.50 (dd, 1H, *J* = 7.8, 1.2 Hz, C3), 7.87 (dd, 1H, *J* = 7.8, 1.4 Hz, C6).

**^13^C NMR** (75 MHz, CDCl_3_) δ 14.1 (C13′, CH_3_), 19.8 (C3′, CH_2_), 22.7 (C12′, CH_2_), 28.7 (C4′, CH_2_), 29.0 (C5′, CH_2_), 29.2 (CH_2_), 29.3 (CH_2_), 29.5 (CH_2_), 29.6 (CH_2_), 29.7 (CH_2_), 31.9 (C11′, CH_2_), 52.0 (C8, CH_3_), 79.2 (C1′), 96.1 (C2′), 124.5 (C2), 127.1 (C5, CH), 130.1 (C6, CH), 131.4 (C4, CH), 132.0 (C1), 134.2 (C3, CH), 167.0 (C7).

**IR** (ATR) $\bar{\upsilon }$*_max_* cm^−1^ 2925, 2853, 2234, 1735, 1718, 1597, 1484, 1447, 1432, 1292, 1275, 1249, 1128, 1083, 756, 701.

**HRMS** (ESI) *m*/*z*: [M+H] Calcd for C_21_H_30_O_2_ 315.2318; Found 315.2312.

#### 2.1.8. Synthesis of Methyl 2-Tridecylbenzoate (**35**)

**34** (142 mg, 0.452 mmol, 1.00 eq.) in MeOH (4 mL) was added to an oven-dried RBF containing PtO_2_.H_2_O (6 mg, 0.023 mmol, 5 mol%). The RBF was next evacuated and then backfilled with H_2_. The reaction mixture was stirred for 1 hr under a H_2_ atmosphere. Following filtration through a celite pad, the solvent was removed in vacuo to afford **35** as a yellow oil (136 mg, 0.428 mmol, 95%).

**^1^H NMR** (400 MHz, CDCl_3_) δ 0.88 (t, 3H, *J* = 6.7 Hz, C13′), 1.16–1.43 (m, 20H, C3′–12′), 1.51–1.65 (m, 2H, C2′), 2.93 (t, 2H, *J* = 7.8 Hz, C1′), 3.87 (s, 3H, C8), 7.16–7.32 (m, 2H, C3, C5), 7.38 (ddd, 1H, *J* = 7.5, 1.5 Hz, C4), 7.84 (dd, 1H, *J* = 7.8, 1.3 Hz, C6).

**^13^C NMR** (100 MHz, CDCl_3_) δ 14.1 (C13′, CH_3_), 22.7 (C12′, CH_2_), 29.4 (CH_2_), 29.5 (CH_2_), 29.6 (CH_2_), 29.7 (CH_2_), 29.7 (CH_2_), 29.7 (CH_2_), 29.8 (CH_2_ × 2), 31.9 (C2′, CH_2_), 32.0 (C11′, CH_2_), 34.5 (C1′, CH_2_), 51.8 (C8, CH_3_), 125.6 (C5, CH), 129.5 (C1), 130.5 (C6, CH), 130.9 (C3, CH), 131.7 (C4, CH), 144.8 (C2), 168.2 (C7).

**IR** (ATR) $\bar{\upsilon }$*_max_* cm^−1^ 3396, 3240, 2853, 1713, 1450, 1432, 1332, 1206, 1139, 1057, 844, 749, 649, 568, 511.

**HRMS** (ESI) *m*/*z*: [M+H] Calcd for C_21_H_34_O_2_ 319.2631; Found 319.2624.

#### 2.1.9. Synthesis of 2-Tridecylbenzoic Acid (**36**)

LiOH (103 mg, 4.280 mmol, 10.00 eq.) was added to an RBF containing methyl 2-tridecylbenzoate (**35**) (136 mg, 0.428 mmol, 1.00 eq.) in THF/MeOH/water 3:1:1 (15 mL). The reaction mixture was heated to reflux for 16 h and then allowed to cool to r.t. before the solvent was removed in vacuo. Following addition of water (15 mL), 2M aqueous HCl (15 mL) was added until the solution reached pH 2, before extraction with diethyl ether (5 × 30 mL). The organic layers were combined, washed with brine and dried over with MgSO_4_. Following filtration, the solvent was removed in vacuo to afford **36** as an off-white solid (123 mg, 0.405 mmol, 94%), mp 32–34 °C.

**^1^H NMR** (400 MHz, CDCl_3_) δ 0.87 (t, 3H, *J* = 6.7 Hz, C13′), 1.18–1.44 (m, 20H, C3′-C12′), 1.55–1.71 (m, 2H, C2′), 3.02 (t, 2H, *J* = 7.8 Hz, C1′), 7.20–7.33 (m, 2H, C3, C5), 7.46 (ddd, 1H, *J* = 7.5, 1.4 Hz, C4), 8.03 (dd, 1H, *J* = 8.2, 1.7 Hz, C6).

**^13^C NMR** (100 MHz, CDCl_3_) δ 14.1 (C13′, CH_3_), 22.7 (C12′, CH_2_), 29.4 (CH_2_), 29.5 (CH_2_), 29.6 (CH_2_), 29.7 (2 × CH_2_), 29.7 (3 × CH_2_), 31.8 (C2′, CH_2_), 31.9 (C11′, CH_2_), 34.6 (C1′, CH_2_), 125.8 (C5, CH), 128.1 (C1), 131.2 (C3, CH), 131.6 (C6, CH), 132.8 (C4, CH), 146.0 (C2), 173.3 (C7).

**IR** (ATR) $\bar{\upsilon }$*_max_* cm^−1^ 2921, 2852, 1689, 1602, 1574, 1456, 1404, 1297, 1266, 928, 747, 721, 657, 561.

**HRMS** (ESI) *m*/*z*: [M+H] Calcd for C_20_H_32_O_2_ 305.2475; Found 305.2476.

#### 2.1.10. General Procedure for the Synthesis of *N*-(Sulfonyl)-2-tridecylbenzamides

2-Tridecylbenzoic acid (**36**) (25 mg, 0.082 mmol, 1.00 eq.) and DMAP (11 mg, 0.090 mmol, 1.10 eq.) were dissolved in dichloromethane (5 mL). The mixture was cooled to 0 °C before addition of the chosen sulfonamide reagent (0.078 mmol, 0.95 eq.). After 15 min, DCC (18 mg, 0.090 mmol, 1.10 eq.) was added and stirring at r.t. was continued for 16 h. The urea byproduct was removed by celite pad filtration, and the solvent was removed in vacuo. The residue was dissolved in diethyl ether (10 mL), poured onto 2M aqueous HCl (20 mL) and extracted with diethyl ether (3 × 30 mL). The organic layers were combined, washed with brine and dried with MgSO_4_. Following filtration, the solvent was removed in vacuo, and the crude residue was subjected to flash chromatography using a suitable eluent.

#### 2.1.11. *N*-(*Tert*-butylsulfonyl)-2-tridecylbenzamide (**37**)

The title compound was synthesised using 2-tridecylbenzoic acid (**36**) (25 mg, 0.082 mmol, 1.00 eq.), DMAP (11 mg, 0.090 mmol, 1.10 eq.), *tert*-butylsulfonamide (11 mg, 0.078 mmol, 0.95 eq.) and DCC (18 mg, 0.090 mmol, 1.10 eq.) in DCM (5 mL). The crude residue was subjected to flash chromatography using hexane-diethyl ether (70:30) to afford **37** as an off-white solid (23 mg, 0.053 mmol, 68%), mp 60–62 °C.

**^1^H NMR** (400 MHz, CDCl_3_) δ 0.88 (t, 3H, *J* = 6.7 Hz, C13′), 1.16–1.38 (m, 20H, C3′-C12′), 1.48–1.68 (m, 2H, overlapped, C2′), 1.54 (s, 9H, C2″–C4″), 2.79 (t, 2H, *J* = 7.9 Hz, C1′), 7.19–7.32 (m, 2H, C3, C5), 7.35–7.49 (m, 2H, C4, C6), 8.11 (s, 1H, N–H).

**^13^C NMR** (100 MHz, CDCl_3_) δ 14.1 (C13′, CH_3_), 22.7 (C12′, CH_2_), 24.5 (C2″–C4″, 3 × CH_3_), 29.4 (CH_2_), 29.5 (CH_2_), 29.6 (CH_2_), 29.6 (4 × CH_2_), 29.7 (CH_2_), 31.9 (C2′, CH_2_), 32.0 (C9′, CH_2_), 33.4 (C1′, CH_2_), 62.4 (C1″), 126.0 (C5, CH), 127.0 (C6, CH), 130.8 (C3, CH), 131.5 (C4, CH), 133.3 (C1), 142.4 (C2), 166.8 (C7).

**IR** (ATR) $\bar{\upsilon }$*_max_* cm^−1^ 3238, 2922, 2852, 1712, 1447, 1429, 1331, 1238, 1138, 1055, 1041, 1019, 843, 749, 649, 567, 511.

**HRMS** (ESI) *m*/*z*: [M+H] Calcd for C_24_H_41_NO_3_S 424.2879; Found 424.2893.

#### 2.1.12. *N*-((4-Chlorophenyl)sulfonyl)-2-tridecylbenzamide (**38**)

The title compound was synthesised using 2-tridecylbenzoic acid (**36**) (25 mg, 0.082 mmol, 1.00 eq.), DMAP (15 mg, 0.090 mmol, 1.10 eq.), 4-chlorobenzenesulfonamide (15 mg, 0.078 mmol, 0.95 eq.) and DCC (18 mg, 0.090 mmol, 1.10 eq.) in DCM (5 mL). The crude residue was subjected to flash chromatography using hexane-diethyl ether (70:30) to afford **38** as an off-white solid (33 mg, 0.069 mmol, 89%), mp 77–78 °C.

**^1^H NMR** (300 MHz, CDCl_3_) δ 0.88 (t, 3H, *J* = 6.7 Hz, C13′), 1.07–1.41 (m, 20H, C3′-C12′), 2.61 (t, 2H, *J* = 7.8 Hz, C1′), 7.15–7.28 (m, 2H, C3, C5), 7.33–7.43 (m, 2H, C4, C6), 7.49–7.59 (m, 2H, C3″, C5″), 8.02–8.14 (m, 2H, C2″, C6″), 8.57 (s, 1H, N–H).

**^13^C NMR** (75 MHz, CDCl_3_) δ 14.1 (C13′, CH_3_), 22.7 (C12′, CH_2_), 29.4 (CH_2_), 29.4 (CH_2_), 26.5 (CH_2_), 29.6 (CH_2_), 29.7 (2 × CH_2_), 29.7 (2 × CH_2_), 31.7 (C2′, CH_2_), 31.9 (C7′, CH_2_), 33.2 (C1′, CH_2_), 126.0 (C5, CH), 127.1 (C6, CH), 129.3 (C3″, C5″, CH), 130.1 (C2″, C6″, CH), 130.9 (C3, CH), 131.8 (C4, CH), 132.0 (C1), 136.9 (C1″), 140.8 (C4″), 142.7 (C2), 166.4 (C7).

**IR** (ATR) $\bar{\upsilon }$*_max_* cm^−1^ 3240, 2923, 2853, 1705, 1586, 1430, 1350, 1241, 1170, 1085, 1057, 890, 848, 753, 616, 568, 484.

**HRMS** (ESI) *m*/*z*: [M+H] Calcd for C_26_H_36_ClNO_3_S 478.2177; Found 478.2172.

### 2.2. Microbiology

**Bacterial strains and culture media.** *X. fastidiosa* Temecula1 was wild-type strain ATCC 700964. The *Xf*DSF-biosensor strain (previously designated X. fastidiosa rpfF*-XfHA biosensor) consists of the rpfF* mutant (E141A E161A) that exhibits blocked DSF synthesis but can still sense externally applied DSF harbouring p*Xf*HA (*hxfA*=::*phoA*) [6]. Inoculum of *X. fastidiosa* was grown on periwinkle wilt GelRite medium (PWG medium) plates for 5 to 7 days before transfer to PD3 broth. All cultures were grown at 28 °C.

**Biofilm assay.** The effects of individual compounds on the biofilm formation capacity of both the wild-type *X. fastidiosa* strain and the X. fastidiosa rpfF* mutant were determined in cells growing in PD3 broth cultures. Cells were grown on PWG plates for 7 days, resuspended in PD3 broth to an OD_600_ of 0.05 and added (2 mL) to PD3 broth cultures containing a final concentration of 10 µM of each compound or an equal volume of DMSO alone as a control. The glass tubes, containing 10 mL of a culture, were shaken (200 rpm) for 24 h at 28 °C, during which time a visible biofilm formed at the liquid–air interface that was attached to the tube. The medium, containing unattached cells, was then removed by aspiration, and the concentration of planktonic cells was assessed as OD_600._ The tubes from which planktonic cells had been removed were washed three times with tap water to remove any unattached cells, and the biomass attached to the glass wall was stained with 2 mL of 1% crystal violet (CV) for 10 min. Excess CV was removed by washing the tubes three times with tap water, and the retained CV was dissolved in 1 mL of 95% ethanol and quantified by measuring absorbance at 595 nm (Spectronic 21D spectrophotometer; Milton Roy, Harvey, LA, USA). The data reported are an average of three replicates for each treatment. The experiments were conducted twice with similar results.

## 3. Results and Discussion

### 3.1. Chemistry

**Figure 2:**
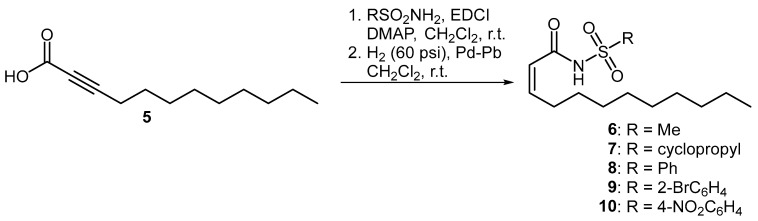
Preparation of olefinic BDSF analogues.

Three series of compounds were evaluated in this study. The first set of molecules were olefinic derivatives of BDSF where a bioisosteric sulfonamide is installed in place of the carboxylic acid functionality. The incorporation of bioisosteric groups in this manner is a well-established strategy for the development of biologically active analogues [15,16,17]. These compounds, which have been shown to disrupt intercellular communication in bacterial species such as *Pseudomonas aeruginosa*, *Stenotrophomonas maltophilia* and *Burkholderia cepacia*, were prepared using a previously published methodology [12,13,18]. Accordingly, propargylic acid **5** was coupled to both alkyl- and aryl-substituted sulfonamides, followed by partial hydrogenation using Lindlar’s catalyst to afford the *cis*-unsaturated 12-carbon *N*-acyl sulfonamides **6**–**10** (Figure 2).

**Figure 3:**
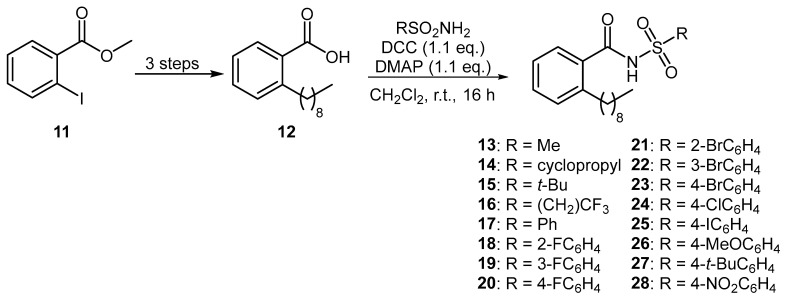
Preparation of aromatic BDSF analogues.

A series of aromatic BDSF analogues were also prepared using an established synthetic route [14]. Starting from methyl 2-iodobenzoate (**11**), common intermediate **12** was prepared over three steps by way of a Sonogashira coupling with 1-nonyne, followed by hydrogenation and subsequent basic hydrolysis (Figure 3). DCC-mediated coupling of carboxylic acid **12** with various sulfonamides produced a diverse range of 16 aromatic *N*-acyl sulfonamides **13**–**28**.

**Figure 4:**
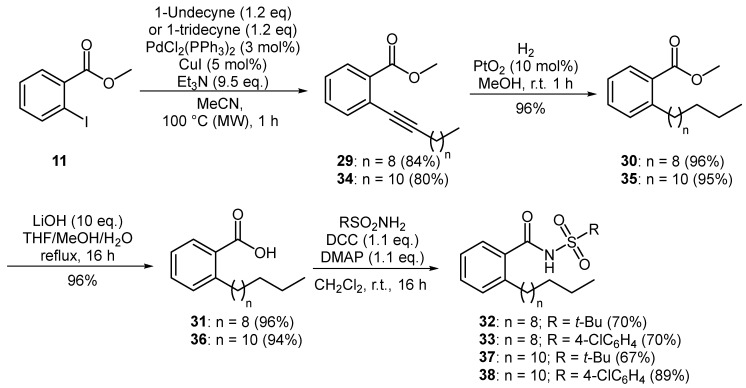
Preparation of aromatic *Xf*DSF analogues.

A similar synthetic strategy was employed for the preparation of novel, longer chain analogues of *Xf*DSF1 and *Xf*DSF2. Sonogashira coupling of aryl iodide **11** with 1-undecyne afforded alkyne **29** in a 84% yield (Figure 4). Based on previous work, it was expected that hydrogenation of **29** at atmospheric pressure for 24 h in the presence of Adam’s catalyst would furnish methyl 2-undecylbenzoate (**30**). However, ^1^H-NMR analysis of the crude reaction mixture revealed the presence of two products in a 34:66 ratio as measured by the respective integrations of the methoxy singlets occurring at 3.88 ppm and 3.65 ppm. While the signals for the minor component matched that of target **30**, the signals for the major product suggested that both the aromatic ring and the alkyne had been reduced. In order to avoid over-reduction, we found that a reaction time of 1 h was sufficient to fully convert alkyne **29** to methyl 2-undecylbenzoate (**30**) in a 96% yield with no evidence of any unwanted side products. Removal of the methyl ester was achieved by treating **30** with excess lithium hydroxide in THF/MeOH/H_2_O at reflux for 16 h, producing pure 2-undecylbenzoic acid (**31**) as a white solid in a 96% yield. Carboxylic acid **31** was coupled to *tert*-butylsulfonamide and 4-chlorobenzenesulfonamide, respectively, using a combination of DCC and DMAP to produce novel *N*-acylsulfonamides **32** and **33** each in a 70% yield. Homologous analogues were produced in a similar manner to furnish *Xf*DSF2 analogues **37** and **38** in yields of 48% and 64% over four steps from 1-tridecyne.

### 3.2. Biological Evaluation

Biofilms are ubiquitous in nature and constitute a key component of microbial survival mechanisms [19,20,21,22]. Quorum sensing-regulated biofilm production also plays a critical role in *X. fastidiosa* adhesion and transmission. The major vectors of *X. fastidiosa* are xylem-fluid-feeding insects such as sharpshooters and spittlebugs [23]. Once ingested, the bacteria multiply in the insect foregut and form a mat-like biofilm [24]. Following initial lateral attachment, the cells align themselves in a polar fashion as the biofilm matures in fully colonised insects [25]. This polar alignment generates a larger surface area which supports the uptake of nutrients [26]. The biofilm plays a vital role in persistence in the host insect. Not only does the matrix facilitate adhesion to the foregut, but it also provides protection from the turbulent environment of the digestive tract. Although extensive bacterial colonisation and biofilm formation is essential for persistence, very few live bacterial cells are required for transmission [27]. Vectors can inoculate plants very quickly after acquisition [28,29]. The insects transmit the pathogen via short hopping flights from plant to plant, which facilitates the rapid spread of disease [30]. Once inside the plant, *X. fastidiosa* begins to multiply and spreads throughout the xylem system from the site of inoculation [31]. The pathogen begins to form biofilm colonies which occupy the bordered pits [32]. In the later stages of infection, the biofilm completely occludes the xylem vessels, restricting water and nutrient transport [33].

Our library of compounds was tested against two different strains of *X. fastidiosa*: wild-type strain Temecula and an rpfF* mutant. The rpfF* mutant has a point mutation that prevents the RpfF protein from producing *Xf*DSF but still permits the involvement of RpfF with RpfC in the perception of *Xf*DSF. Given that the wild-type strain would produce *Xf*DSF1 and *Xf*DSF2 and their presence would have made it difficult to disentangle any agonistic effect that may have been observed with our compounds in such cultures, agonistic effects could instead be observed by examining the behaviour of the rpfF* mutant. By contrast, antagonism of *Xf*DSF signalling in *X. fastidiosa* was discernible in the wild-type strain.

Bacterial cultures were grown on periwinkle wilt GelRite (PWG) agar as *X. fastidiosa* produces less *Xf*DSF on this medium. Harvested cells were dispersed into a Pierce’s disease (PD3) broth and shaken vigorously for four days. Compounds were added at a final concentration of 10 µM. The amount of DMSO used to solubilise the compounds was kept to an absolute minimum, with a final concentration of DMSO in the various cultures of 0.05%. Pure DMSO was also included as a control. The initial cell concentration of each of the strains was approximately 10^7^ cells/mL as determined by the optical density measured at a wavelength of 600 nm (OD_600_). After four days, substantial growth of most of the cultures had occurred. At this point, many of the cells had formed a biofilm ring at the air/medium interface.

**Figure 5:**
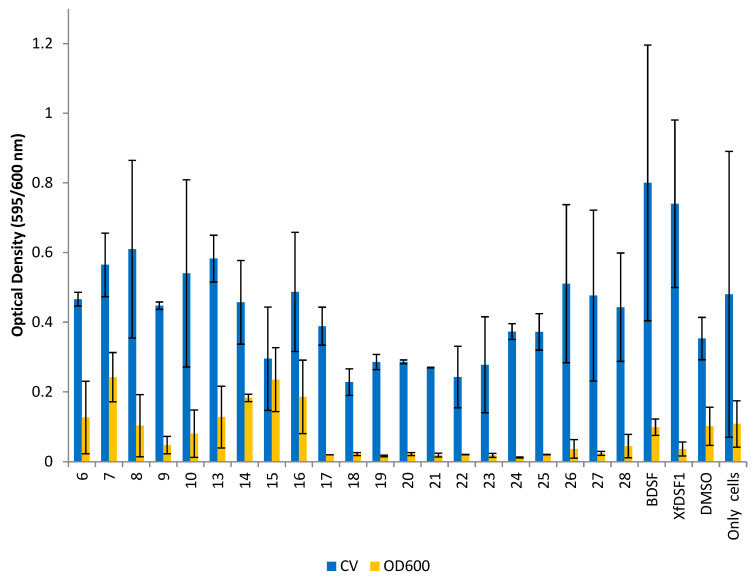
**Optical density readings for wild-type strain Temecula.** OD_595_ abundance of crystal violet retained in biofilm cells attached to glass culture tubes after addition of BDSF analogues (blue bars). OD_600_ abundance of planktonic cells remaining in suspension after addition of BDSF analogues (yellow bars). The error bars represent the standard deviations of the means.

**Figure 6:**
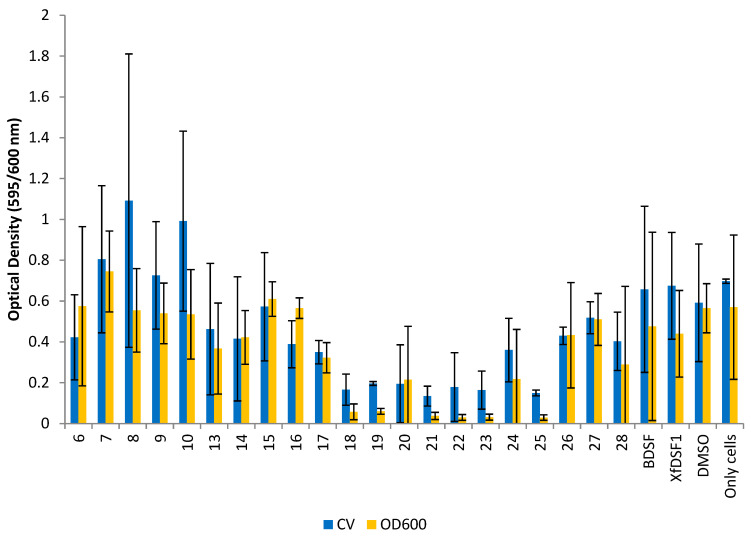
Optical density readings for rpfF* mutant. OD_595_ abundance of crystal violet retained in biofilm cells attached to glass culture tubes after addition of BDSF analogues (blue bars). OD_600_ abundance of planktonic cells remaining in suspension after addition of BDSF analogues (yellow bars). The error bars represent the standard deviations of the means.

The planktonic cells remaining in solution were carefully removed and their abundance determined by again measuring the OD_600_ of these cells. A crystal violet (CV) binding assay was used to determine the abundance of biofilm biomass in each culture. The optical density conferred by the bound crystal violet was measured which was proportional to the number of cells in the biofilm. Measures of the planktonic and biofilm cells (OD_600_ and CV, respectively) are shown separately for the wild-type strain (Figure 5) and the rpfF* mutant (Figure 6). It should be noted that the biofilms produced by *X. fastidiosa* are not particularly adhesive to glass surfaces, and there tends to be a relatively large variation in the proportion of the biofilm that ends up adhering to the glass tube surface (as opposed to that remaining as clumped cells in the culture)—leading to the relatively large variation in biofilm abundance seen in such experiments where crystal violet staining of wall-associated cells is assessed.

Analogues **6**–**10** and **13**–**28** were initially evaluated. Compounds **6**–**10** all feature a 12-carbon chain, in addition to a *cis*-unsaturated double bond, and can be considered first generation, direct mimics of BDSF (**2**). The original carboxylic acid is replaced with either an aliphatic (**6**–**7**) or aromatic (**8**–**10**) *N*-acyl sulfonamide bioisostere. As *cis*-unsaturated fatty acids are prone to isomerisation, molecules **13**–**28** represent a second generation series of BDSF analogues whereby a rigid benzene ring is installed to permanently lock the molecular geometry [34]. This series incorporates a wider range of aliphatic (**13**–**16**) and aromatic (**17**–**28**) sulfonamide substituents. The data in Figure 5 suggest that many of our compounds exhibit an inhibitory effect on both planktonic cell growth and biofilm biomass accumulation in the wild-type Temecula strain. In the CV assay, *ortho*-fluorophenylsulfonamide **18** (OD 0.228), *ortho*-bromophenylsulfonamide **21** (OD 0.269) and *meta*-bromophenylsulfonamide **22** (OD 0.243) produced the strongest inhibitory effect on biofilm biomass accumulation compared to the DMSO control (OD 0.353). A clear structure activity trend emerged with other haloaryl-substituted sulfonamides also inhibiting biofilm production, including *meta*-fluorosulfonamide **19** (OD 0.286), *para*-fluorosulfonamide **20** (OD 0.286) and *para*-bromosulfonamide **23** (OD 0.278). By contrast, all of the olefinic analogues **6**–**10**, as well as signalling molecules BDSF (**2**) and *Xf*DSF1 (**3**), produced OD readings greater than the DMSO control. Among the aromatic analogues, methylsulfonamide **13**, cyclopropylsulfonamide **14** and 3,3,3-trifluoropropylsulfonamide **16**, as well as *para*-methoxyphenylsulfonamide **26**, *para*-*tert*-butylsulfonamide **27** and *para*-nitrophenylsulfonamide **28**, were among the strongest promoters of biofilm cell accumulation. These latter results highlight how modifying the phenyl ring substituents can switch activity from biofilm inhibition to biofilm promotion.

The OD_600_ readings measuring the planktonic cell growth in Figure 5 revealed that all of our aromatic analogues containing an arylsulfonamide (**17**–**28**) displayed strong growth inhibition compared to the control. Of these, *para*-chlorophenylsulfonamide **24** was the most active with an OD reading of 0.012, an 8-fold reduction compared to the DMSO control (OD 0.102). Only a small negative effect of DMSO on cell growth was noted. The arylsulfonamides together had a mean OD reading of 0.022, representing a 4-fold average reduction in planktonic cell growth. While *para*-methoxyphenylsulfonamide **26** (OD 0.037) and *para*-nitrophenylsulfonamide **28** (OD 0.045) were slightly less active, they were still comparable to *Xf*DSF1 (**3**) (OD 0.036). The alkyl-substituted sulfonamides **13**–**16** did not display an inhibitory effect. Of the olefinic analogues, *ortho*-bromophenylsulfonamide **9** (OD 0.048) was the only candidate to inhibit growth to a notable level.

The rpfF* mutant is often used as a measure of agonistic effects on cell growth since endogenous DSF is not present. The use of the rpfF* mutant was especially important in this study as the effects of particular DSF analogues on phenotypes of *Xylella fastidiosa* were being assessed. The addition of such analogues to strains that were capable of producing their own native DSF would have complicated interpretation of the assessment of the direct effects of the compounds on *X. fastidiosa* phenotypes such as biofilm formation. The behaviour of our compounds towards the rpfF* mutant strain was similar to the wild-type strain, with a reduction in cell growth observed in most cultures (Figure 6). The CV and OD_600_ readings for the DMSO control were recorded as 0.592 and 0.565, respectively—reflecting the minor inhibitory effect of DMSO on cell growth. The optical density readings from the CV assay revealed that 17 of the 21 compounds reduced biofilm biomass accumulation. Of the aromatic analogues, *ortho*-bromophenylsulfonamide **21** was the most potent inhibitor, producing an OD reading of 0.135, a 4-fold reduction compared to the control. Other haloaryl-substituted sulfonamides, including *ortho*-fluorosulfonamide **18** (OD 0.166), *para*-bromosulfonamide **23** (OD 0.164) and *para*-iodosulfonamide **25** (OD 0.150), were almost equally effective, reflecting the same trend observed with Temecula. By contrast, alkyl-substituted sulfonamides **13**–**16** and non-halogenated arylsulfonamides **26**–**28** exhibited poor biofilm inhibition in the rpfF* mutant. Methylsulfonamide **6** (OD 0.423) was the only olefinic analogue to reduce biofilm biomass accumulation, while olefinic phenylsulfonamide **8** (OD 1.092) produced the largest growth enhancement effect with a 1.8-fold increase. Signalling molecules BDSF (**2**) (OD 0.658) and *Xf*DSF1 (**3**) (OD 0.675) also increased biofilm biomass accumulation in the rpfF* strain.

The most potent compound in the OD_600_ assay was *para*-iodophenylsulfonamide **25** (OD 0.029) which was responsible for an almost 19-fold reduction in planktonic cell growth. Once again, the halogen-substituted sulfonamides were among the most potent inhibitors with an average OD reading of 0.072, representing a 7-fold growth reduction, observed in the cultures containing fluoro- and bromo-substituted phenylsulfonamides **18**–**20** and **21**–**23**, respectively. While the olefinic analogues **6**–**10** were ineffective inhibitors, only cyclopropylsulfonamide **7** (OD 0.745) actually increased planktonic cell growth in this strain. In line with expectations, BDSF (**2**) and *Xf*DSF1 (**3**) increased biofilm biomass accumulation in the CV assay but reduced planktonic cell growth with OD_600_ readings of 0.476 and 0.440, respectively. It is worth noting that BDSF analogues **6**–**9** and **13**–**28** have been previously assessed using a combination of MTT assays [13] and the *Galleria mellonella* in vivo model [14] and no compound in this series was found to display significant cytotoxic effects.

**Figure 7:**
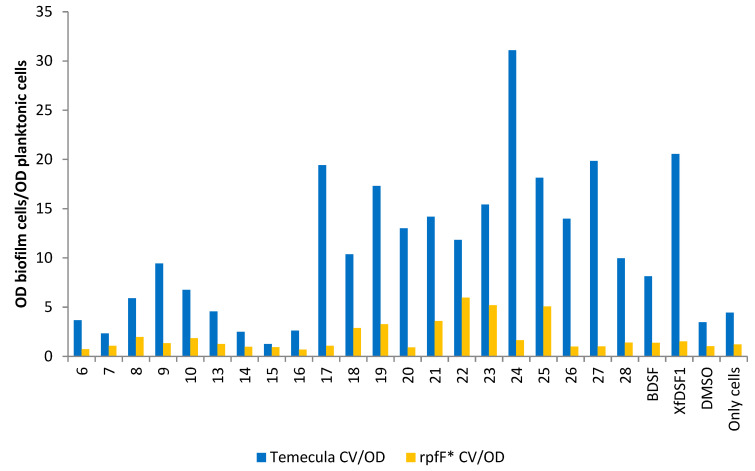
Ratio of the biofilm cells to planktonic cells from cultures of wild-type strain Temecula and rpfF* mutant. Shown is the ratio of OD_595_ reflecting the abundance of crystal violet retained in biofilm cells attached to glass culture tubes relative to the OD_600_ of planktonic cells remaining in suspension after addition of DSF analogues to cultures of wild-type *X. fastdiosa* (blue bars) or an rpfF* mutant (yellow bars).

One means of identifying agonistic and antagonistic effects in *X*. *fastidiosa* is to examine the ratio of the biofilm biomass to planktonic cells in each culture, i.e., the ratio of CV to OD_600_ optical densities (Figure 7). For almost all of our compounds, including for the positive controls of BDSF (**2**) and *Xf*DSF1 (**3**) signalling molecules, stronger biofilm formation was observed in the wild-type cultures compared to rpfF*. This most likely reflects the accumulation of native DSF that facilitates biofilm formation and which is made only by the WT strain and not the rpfF* mutant. The stronger induction of biofilm by *Xf*DSF1 (**3**) in the wild-type strain compared to rpfF* mutant suggested that the assay was working as expected, but also suggested that the endogenous (intracellular) production of DSF by the cells results in higher concentrations of this signal molecule than when supplied extracellularly. That is, there may be limits to the solubility of these molecules or their ability to be taken up from the extracellular milieu. In the presence of phenylsulfonamide **17**, *para*-chlorophenylsulfonamide **24** and *tert*-butylphenylsulfonamide **27**, there was an approximately 18-fold difference between the CV/OD ratios in the wild-type Temecula and rpfF* strains. The smallest difference in CV/OD ratios for the Temecula and rpfF* strains was recorded for *tert*-butylsulfonamide **15**.

In general, *X. fastidiosa* is more sensitive to longer chain DSF signals compared to other bacterial species. These signalling molecules usually range from 12 to 18 carbons [6]. The most active of these quorum sensing signals is 16-carbon *Xf*DSF2 (**4**) (Figure 1). We originally designed our aromatic *N*-acyl sulfonamides **13**–**28** as analogues of 12-carbon BDSF (**2**). As some of our aromatic analogues were active as either agonists or antagonists, we wondered if extending the alkyl chain at the C2 position would give rise to a more pronounced biological effect. This second series of compounds, containing 14-carbon or 16-carbon backbones, could serve as analogues of *Xf*DSF1 (**3**) or *Xf*DSF2 (**4**), respectively. The longer chain analogues, namely *tert*-butylsulfonamides **32** and **37** and *para*-chlorophenylsulfonamides **33** and **38**, as well as our *Xf*DSF1 (**3**) and *Xf*DSF2 (**4**) reference samples, were evaluated using the same CV and OD_600_ assays described previously.

**Figure 8:**
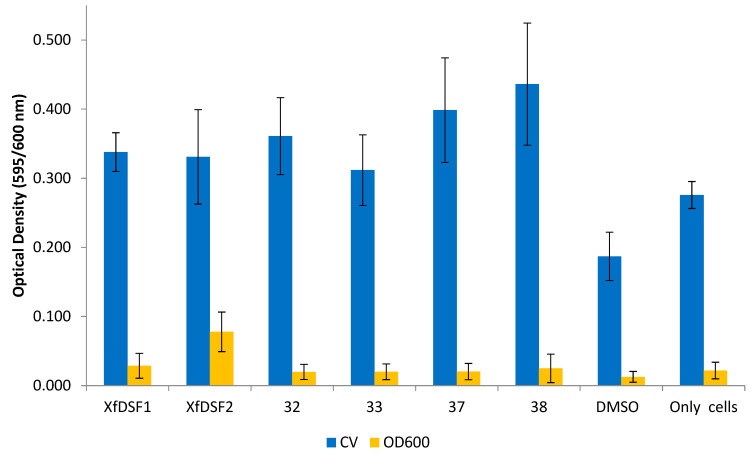
**Optical density readings of longer chain *Xf*DSF analogues for wild-type strain Temecula.** OD_595_ abundance of crystal violet retained in biofilm cells attached to glass culture tubes after addition of *Xf*DSF analogues (blue bars). OD_600_ abundance of planktonic cells remaining in suspension after addition of *Xf*DSF analogues (yellow bars). The error bars represent the standard deviations of the means.

In contrast to the 2-nonyl-substituted analogues **13**–**28**, the longer chain analogues appeared to increase both biofilm biomass and planktonic cell growth in the wild-type Temecula strain (Figure 8). In the CV assay, all of the bioisosteres **32**–**33**, **37**–**38,** in addition to signalling molecules *Xf*DSF1 (**3**) and *Xf*DSF2 (**4**), produced OD readings which were almost double that of the DMSO control. The 2-undecyl derivatives, namely *tert*-butylsulfonamide **32** (0.361) and *para*-chlorophenylsulfonamide **33** (0.312), were comparable to signalling molecules *Xf*DSF1 (0.338) and *Xf*DSF2 (0.331). The longer 2-tridecyl analogues were more active than their 2-undecyl counterparts—*tert*-butylsulfonamide **37** (0.399) and *para*-chlorophenylsulfonamide **38** (0.436) caused 2.2- and 2.4-fold increases in biofilm biomass growth, respectively. In the OD_600_ assay measuring planktonic cell growth, most compounds displayed comparable activity to the DMSO control (0.013). One molecule which did enhance cell growth was *Xf*DSF2 (0.078). We had expected that extending the chain length at the C-2 position would enhance the inhibitory activity of our analogues. These results appear to contradict this hypothesis.

**Figure 9:**
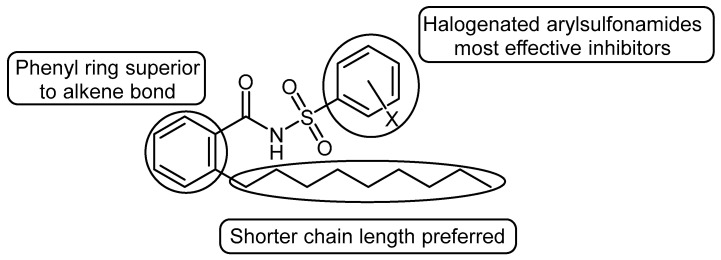
Major structure–activity relationships.

Figure 9 summarises the main structure–activity relationships identified in this study. The most effective biofilm inhibitors were found to be the aromatic BDSF analogues, in particular those incorporating haloaryl-substituted sulfonamides. The corresponding olefinic analogues tended to promote, rather than inhibit, biofilm production in *X. fastidiosa*. Extending the chain length in the equivalent aromatic *Xf*DSF1 or *Xf*DSF2 bioisosteres was not favourable, and resulted in significantly reduced biological activity, contrary to our initial expectations. Although determination of the mode-of-action was not a goal of this study, it seems likely that our bioisosteric analogues, acting as DSF mimics, behave like DSF signals and interact with a variety of downstream regulators such as RpfC and RpfG. It is known that *Xf*DSF is required for de-repression of *rpfC* in the presence of RpfF [8]. Furthermore, RpfC is also involved in a signal cascade involving RpfG and other regulators that determine the intracellular concentration of second messengers such as cyclic di-GMP that ultimately determine phenotypes such as the expression of cellular adhesins important in the biofilm formation and virulence of this pathogen.

## 4. Conclusions

In recent years, *X. fastidiosa* has been the subject of increased focus as a result of its significant economic impact. Losses of vines from Pierce’s disease and associated preventative measures cost the Californian wine-making industry USD 104 million per year [35], while, in Brazil, citrus variegated chlorosis has resulted in the loss of millions of citrus trees at a cost of USD 120 million per year [36]. In October 2013, scientists reported that *X. fastidiosa* was responsible for OQDS occurring in the southern Italian region of Apulia [37]. Over a three year period, OQDS was responsible for losses up to EUR 390 million in olive oil production in Italy [38]. The bacterium now threatens to become a significant plant pest throughout the Mediterranean basin. According to a JRC report, the microbe could cost the European Union up to EUR 5.5 billion per year, and consumers will likely be impacted in the form of higher prices [39,40].

DSF signalling molecules play a key role in regulating biofilm formation in *X. fastidiosa*. The production of biofilm is, in turn, intrinsically linked with cell adhesion and transmission. Accordingly, the development of DSF analogues may constitute a useful strategy for *Xylella* control by disrupting its innate ability to form adhesive biofilms. In this paper, we have demonstrated that *N*-acyl sulfonamide derivatives of BDSF can either inhibit or promote biofilm formation in *X. fastidiosa* depending on the nature of the sulfonamide substituent. In particular, haloaryl-substituted sulfonamides were found to be effective biofilm inhibitors. In general, shorter chain analogues of BDSF proved to be more potent, while longer chain derivatives of *Xf*DSF1 and *Xf*DSF2 prompted a reduced biological response. These results demonstrate that control of biofilm formation in *X. fastidiosa* can be influenced by the addition of appropriately designed DSF analogues. The strategy outlined here may provide opportunities for the control of this important plant pathogen.

## Data Availability

The original contributions presented in the study are included in the article/Supplementary Material, further inquiries can be directed to the corresponding authors.

## Supplementary Materials

The following supporting information can be downloaded at: https://www.mdpi.com/article/10.3390/microorganisms12122496/s1, Compound Spectra.

## Abbreviations

BDSF	*Burkholderia* diffusible signal factor
DCC	*N*,*N*′-Dicyclohexylcarbodiimide
DCM	Dichloromethane
DMAP	Dimethylaminopyridine
DMF	Dimethylformamide
DMSO	Dimethyl sulfoxide
DSF	Diffusible signal factor
OD	Optical density
OQDS	Olive quick decline syndrome (OQDS)
QS	Quorum sensing
RBF	Round bottom flask
r.t.	Room temperature
WT	Wild-type
*Xf*DSF	*X. fastidiosa* diffusible signal factor
