# Supplementary material for: BDSF Analogues Inhibit Quorum Sensing-Regulated Biofilm Production in Xylella fastidiosa
Source: Microorganisms. 2024 Dec 4;12(12):2496. doi: 10.3390/microorganisms12122496 (PMC11727845; doi:10.3390/microorganisms12122496)

Supplementary Information

BDSF analogues inhibit quorum sensing-regulated biofilm production in Xylella fastidiosa

Conor Horgan[1,2,3]*, Clelia Baccari[4]*, Michelle O'Driscoll[1,2,3], Steven E. Lindow[4#] and Timothy P. O'Sullivan[1,2,3#].

Affiliation

[1]School of Chemistry, University College Cork, Cork, Ireland; [2]School of Pharmacy, University College Cork, Cork, Ireland; [3]Analytical and Biological Chemistry Research Facility, University College Cork, Cork, Ireland; [4]Department of Plant and Microbial Biology, University of California, Berkeley, CA 94720, USA.

Methyl 2-(Undec-1-yn-1-yl)benzoate (29)

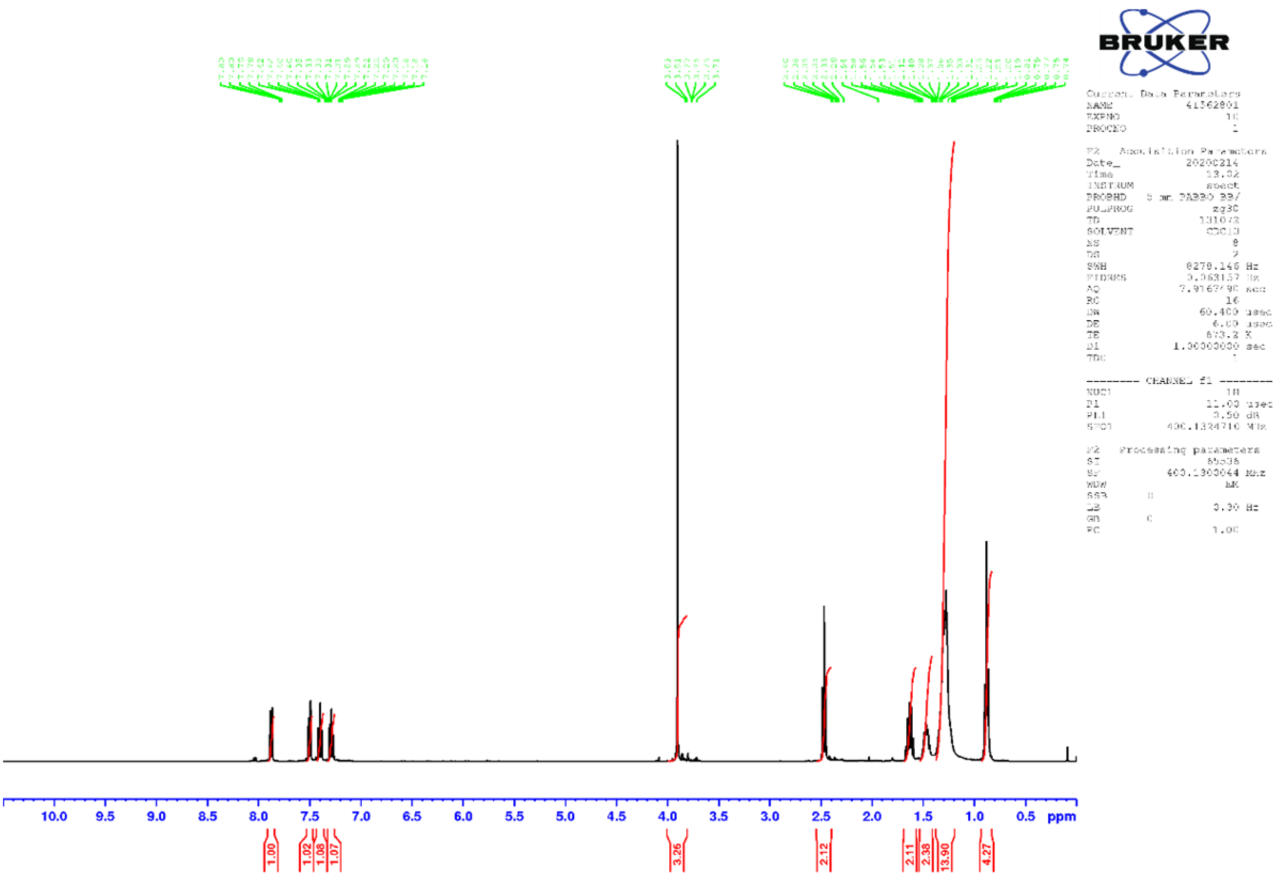

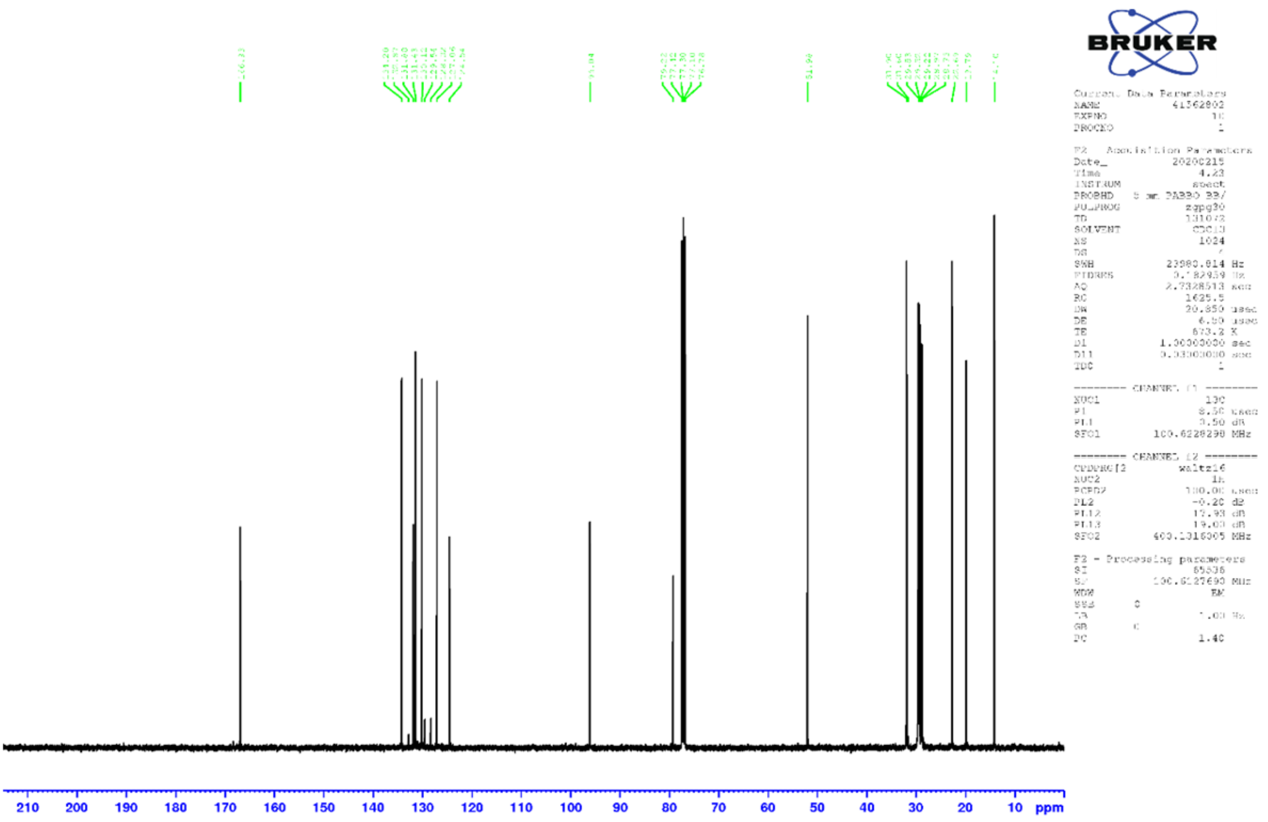

Methyl 2-Undecylbenzoate (30)

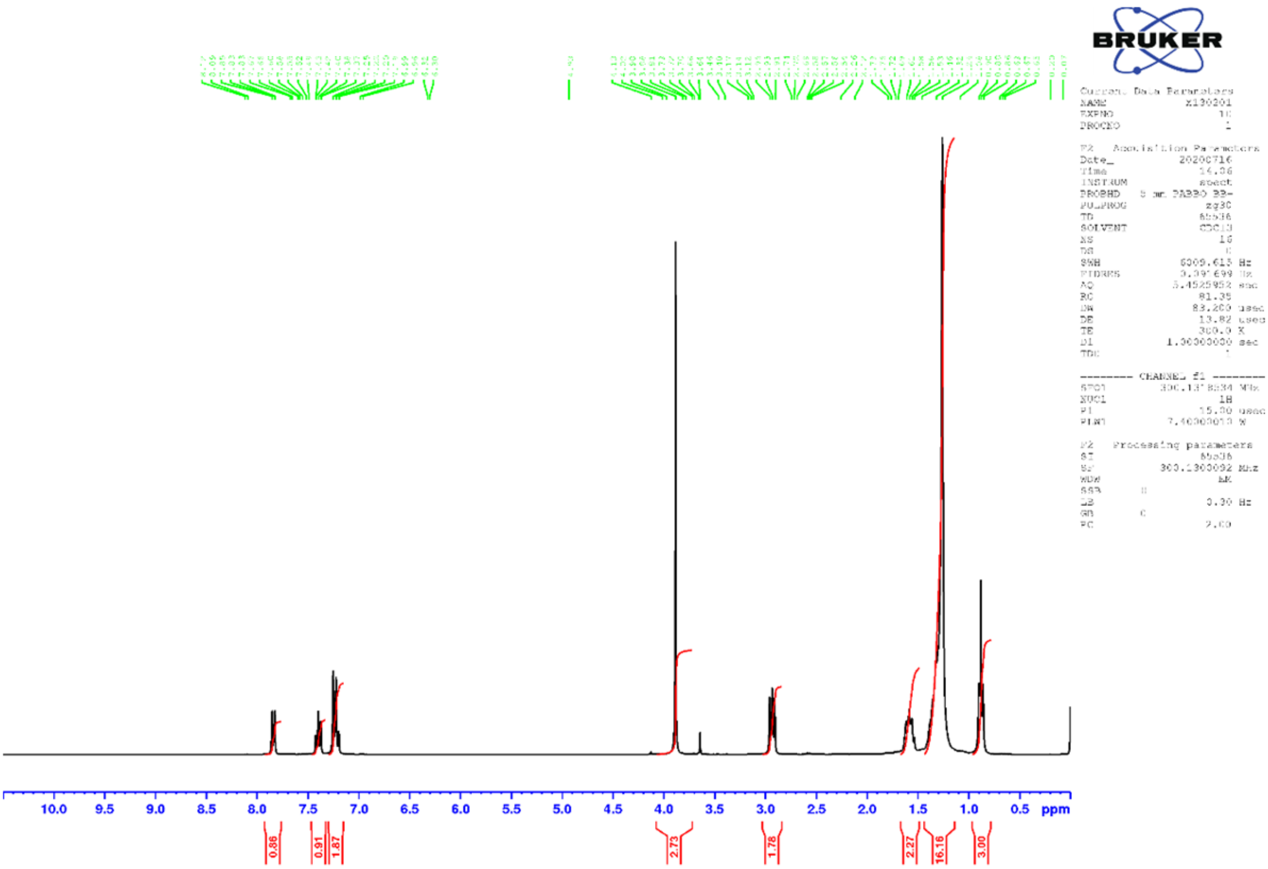

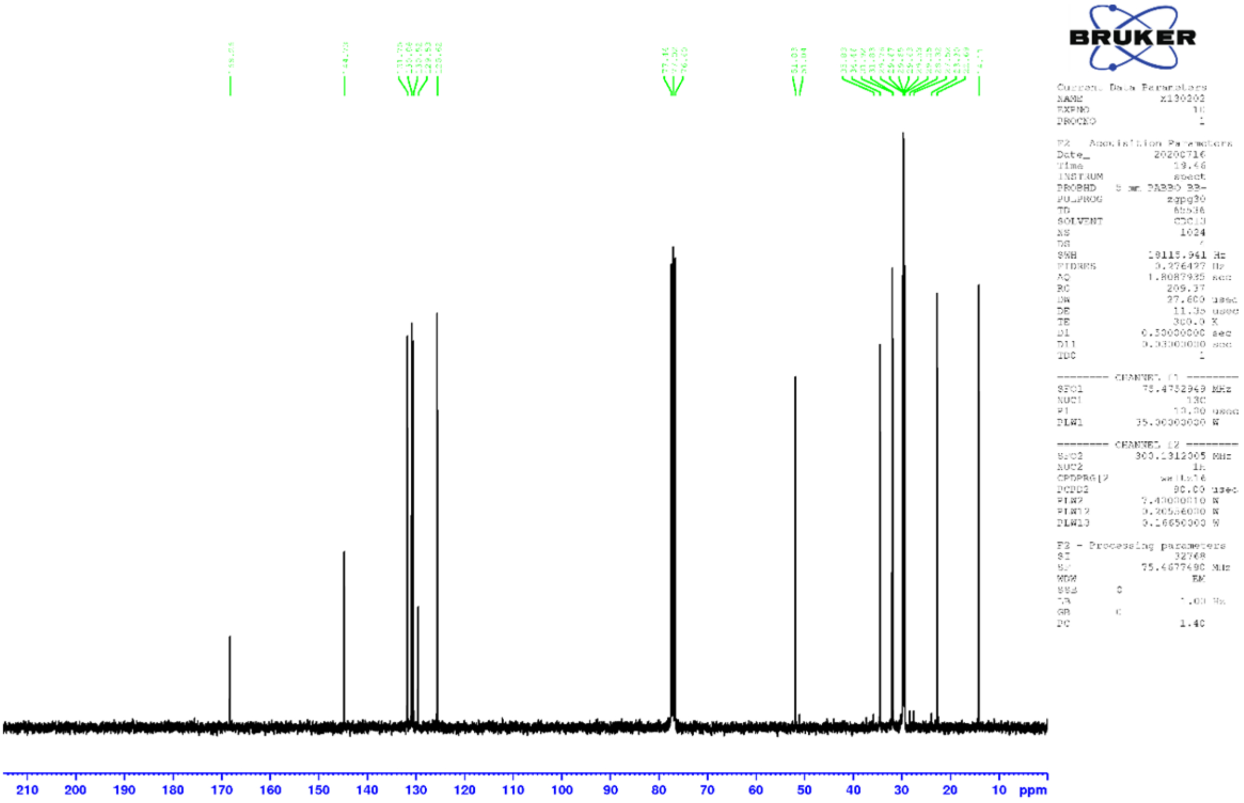

2-Undecylbenzoic Acid (31)

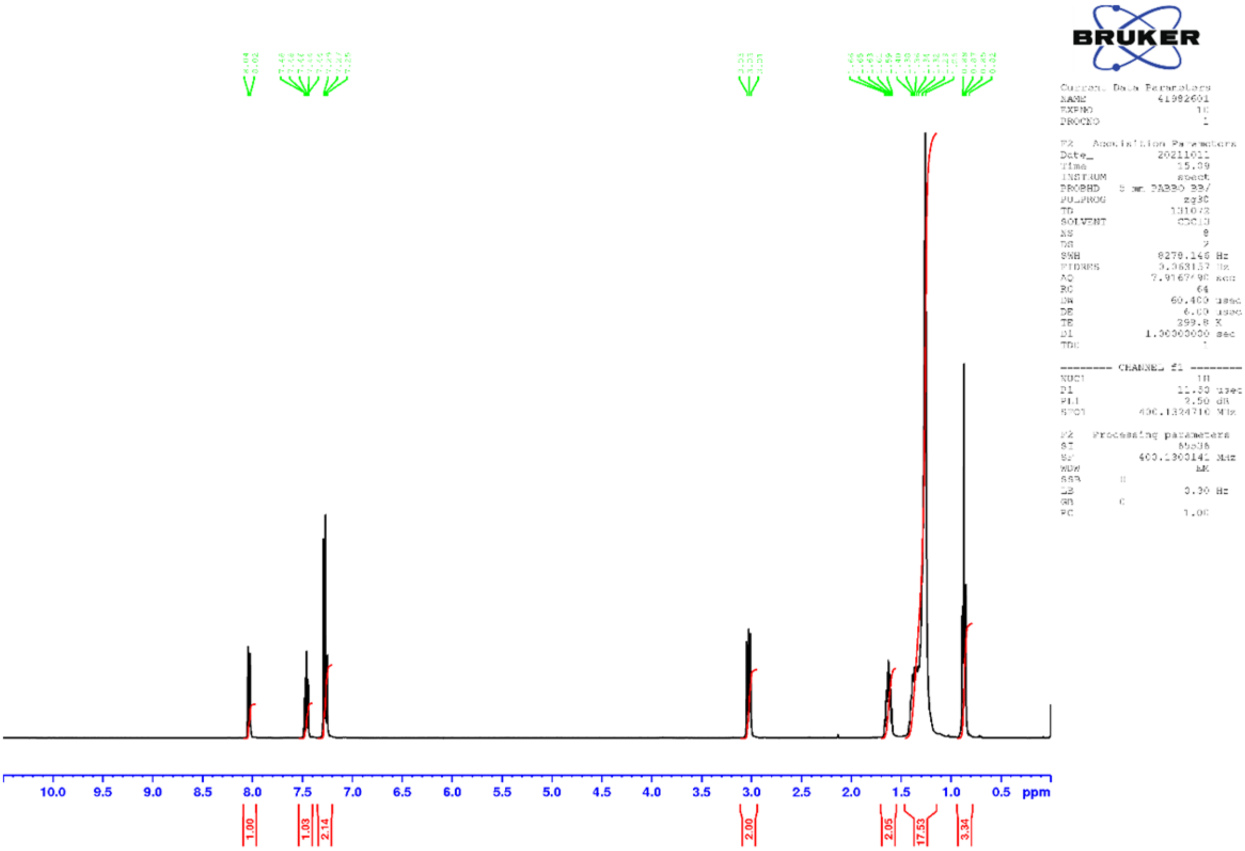

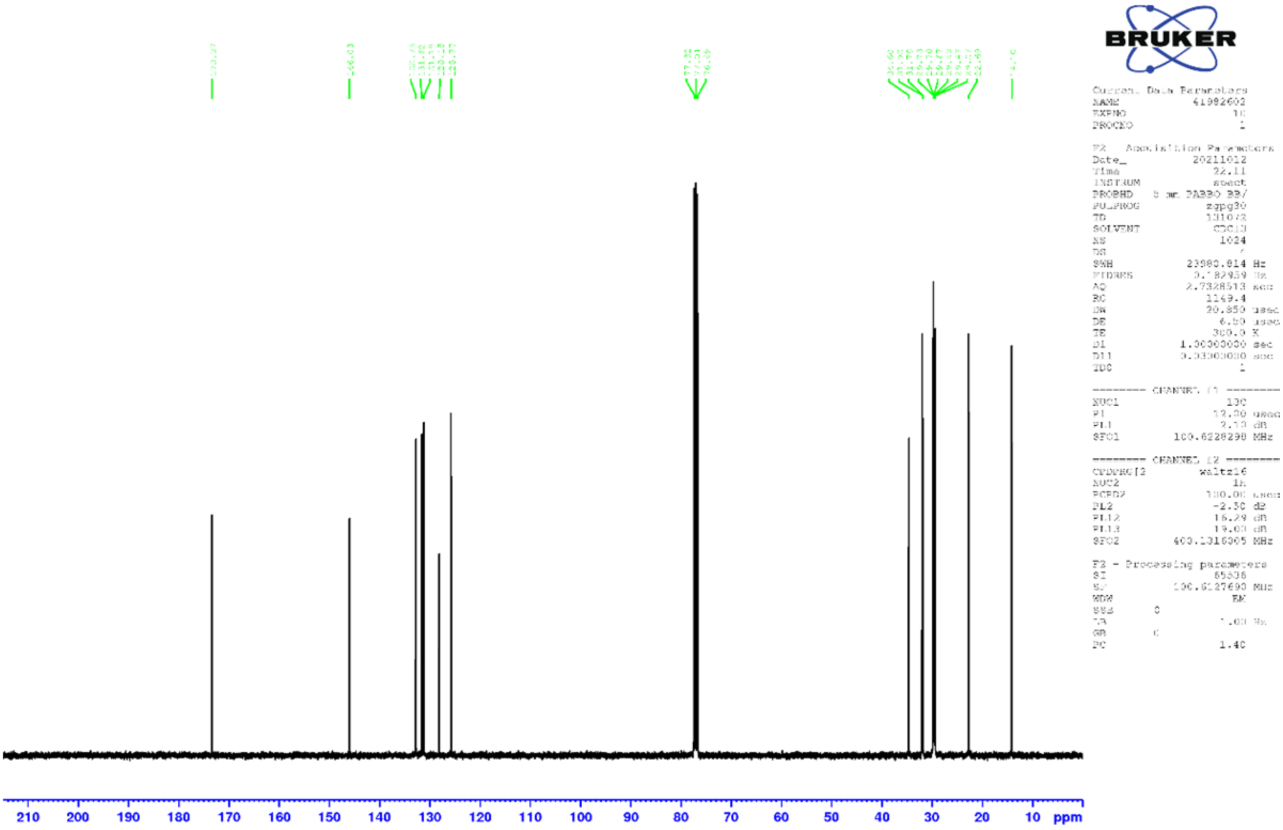

N-(*tert*-Butylsulfonyl)-2-undecylbenzamide (32)

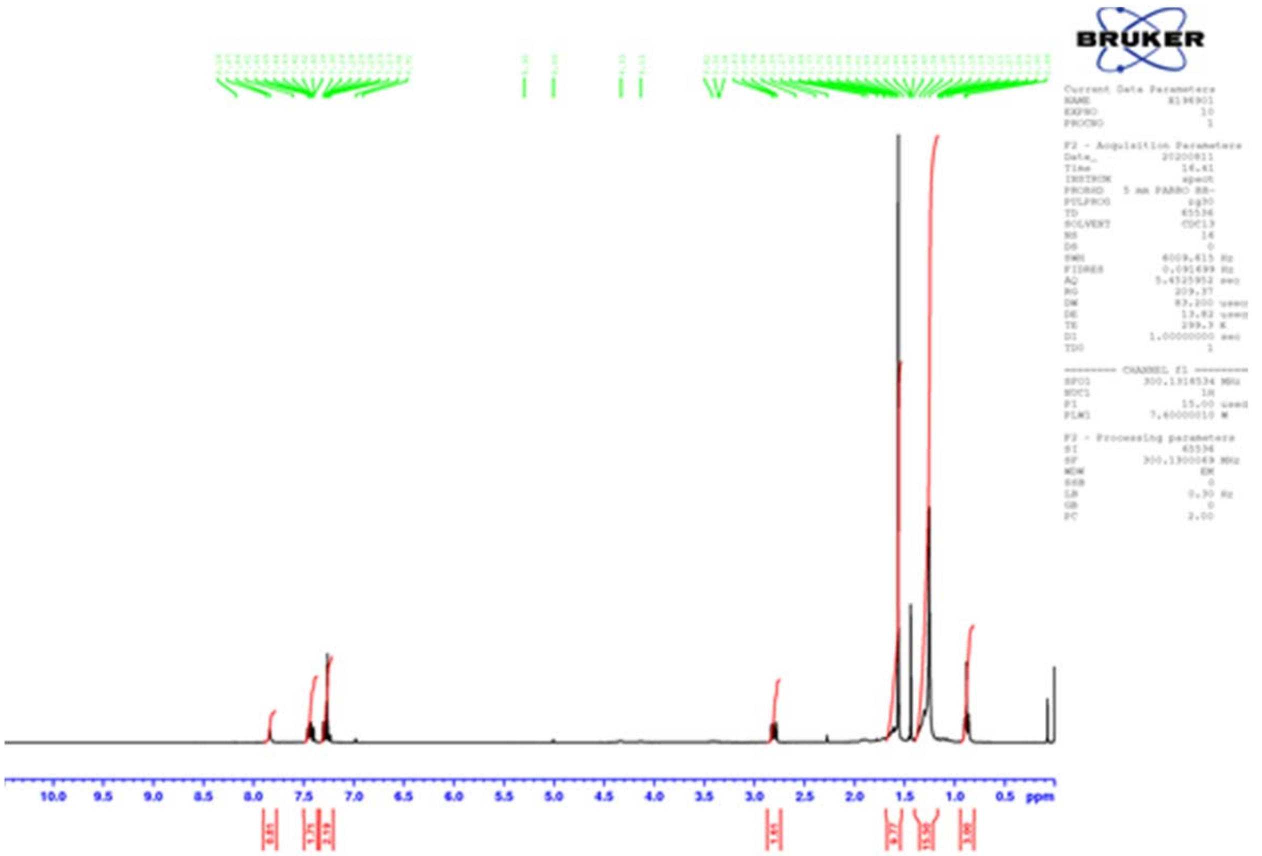

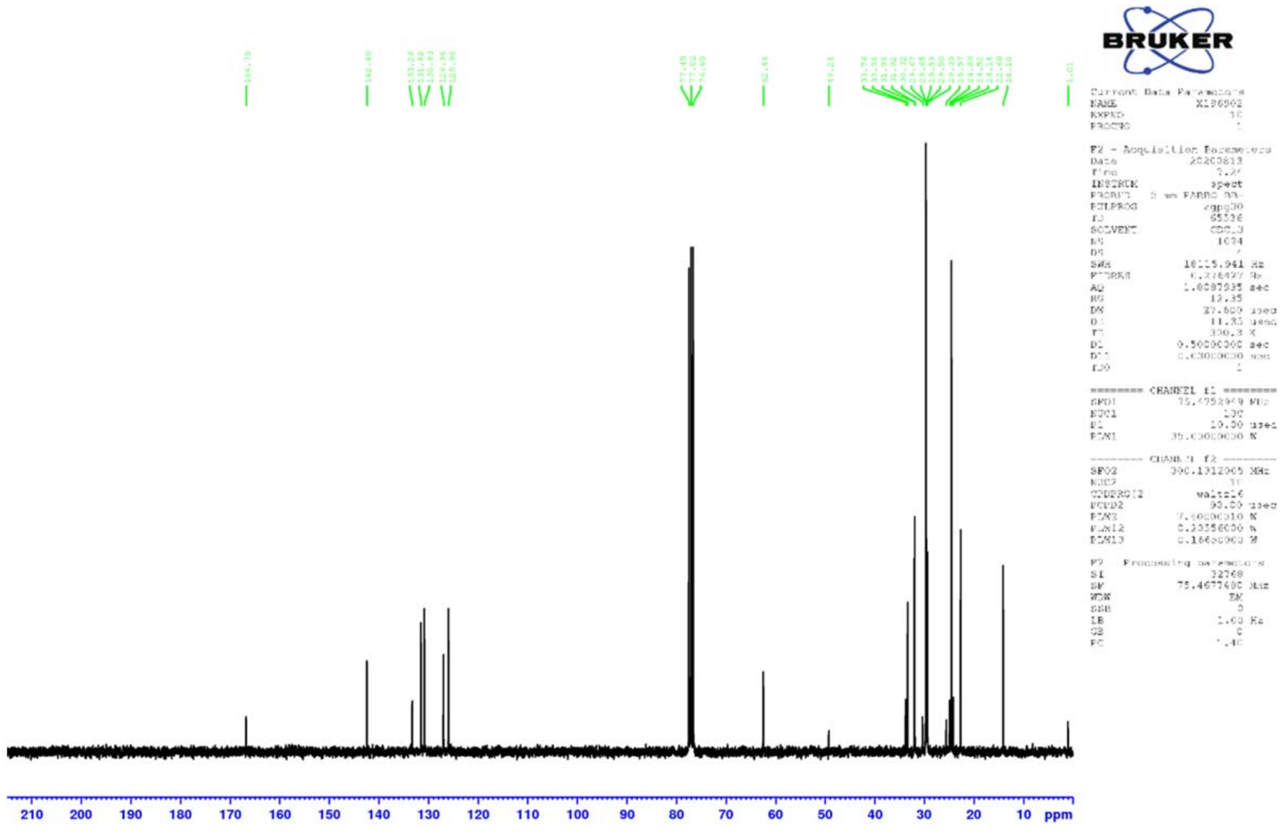

N-((4-Chlorophenyl)sulfonyl)-2-undecylbenzamide (33)

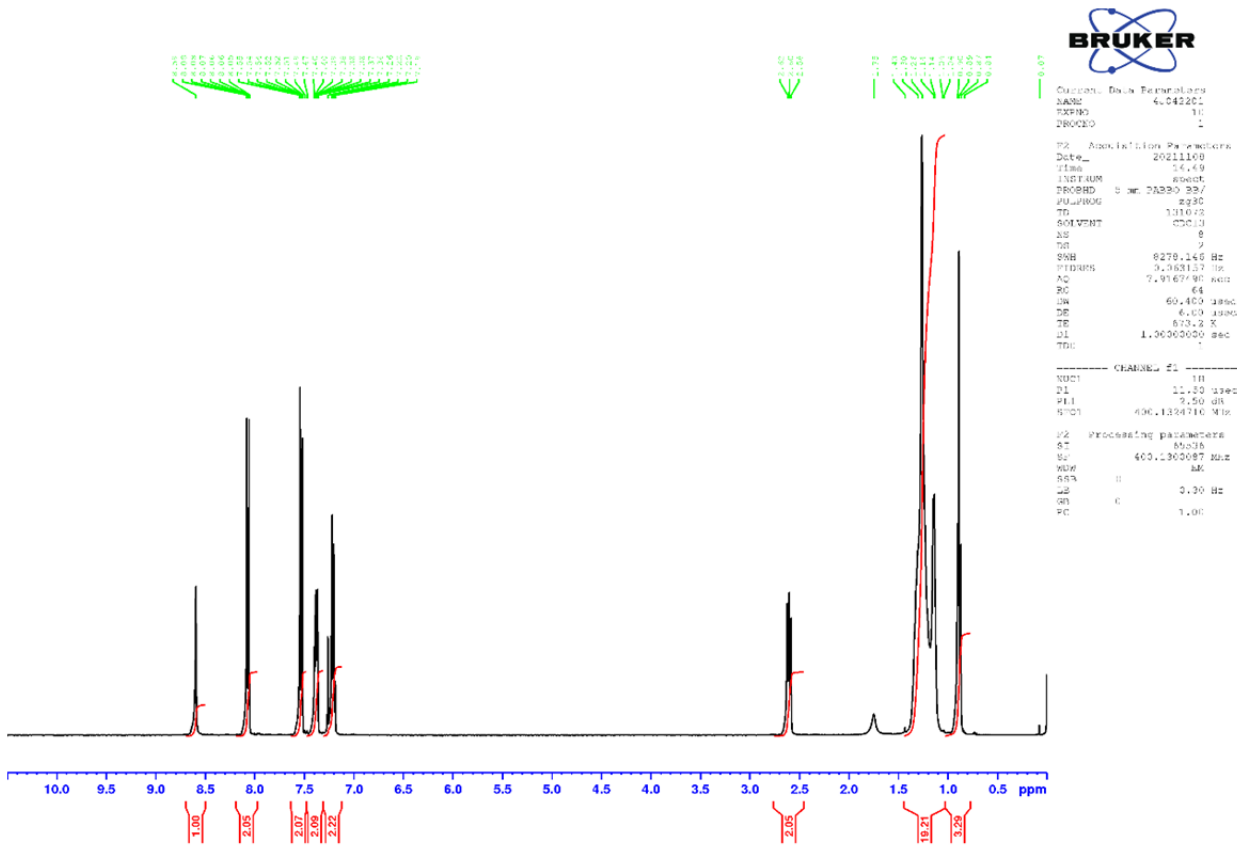

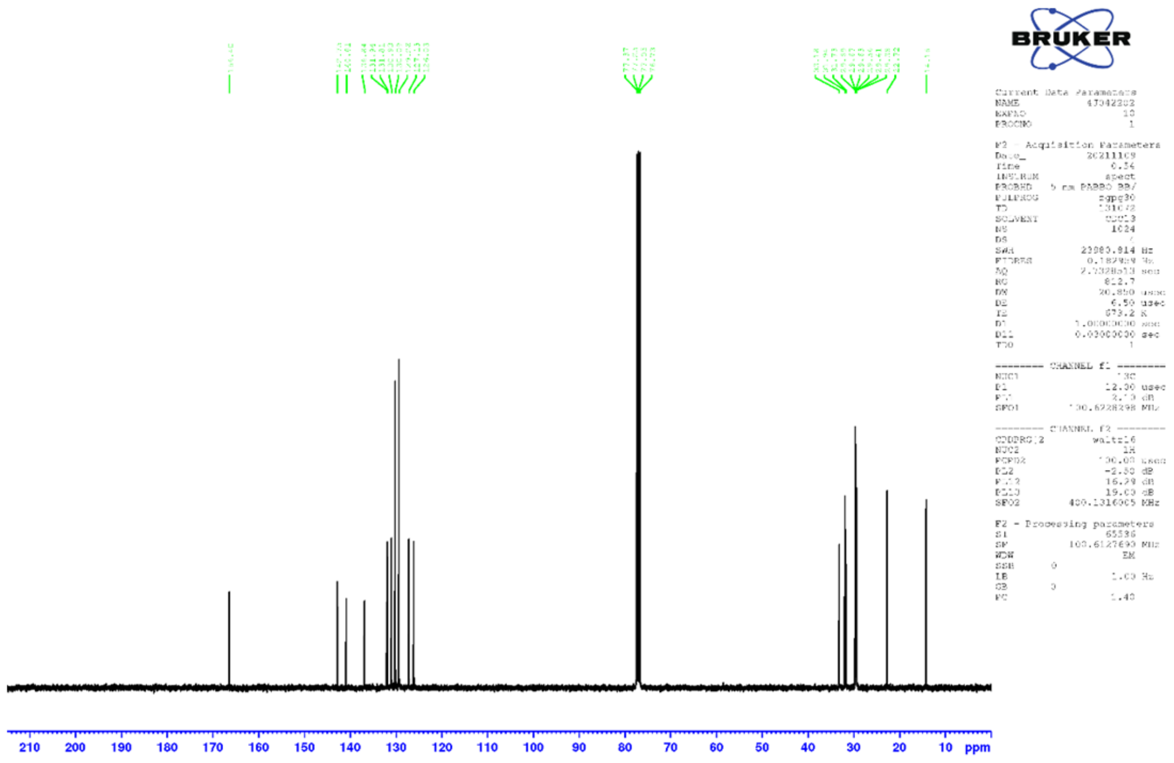

Methyl 2-(Tridec-1-yn-1-yl)benzoate (34)

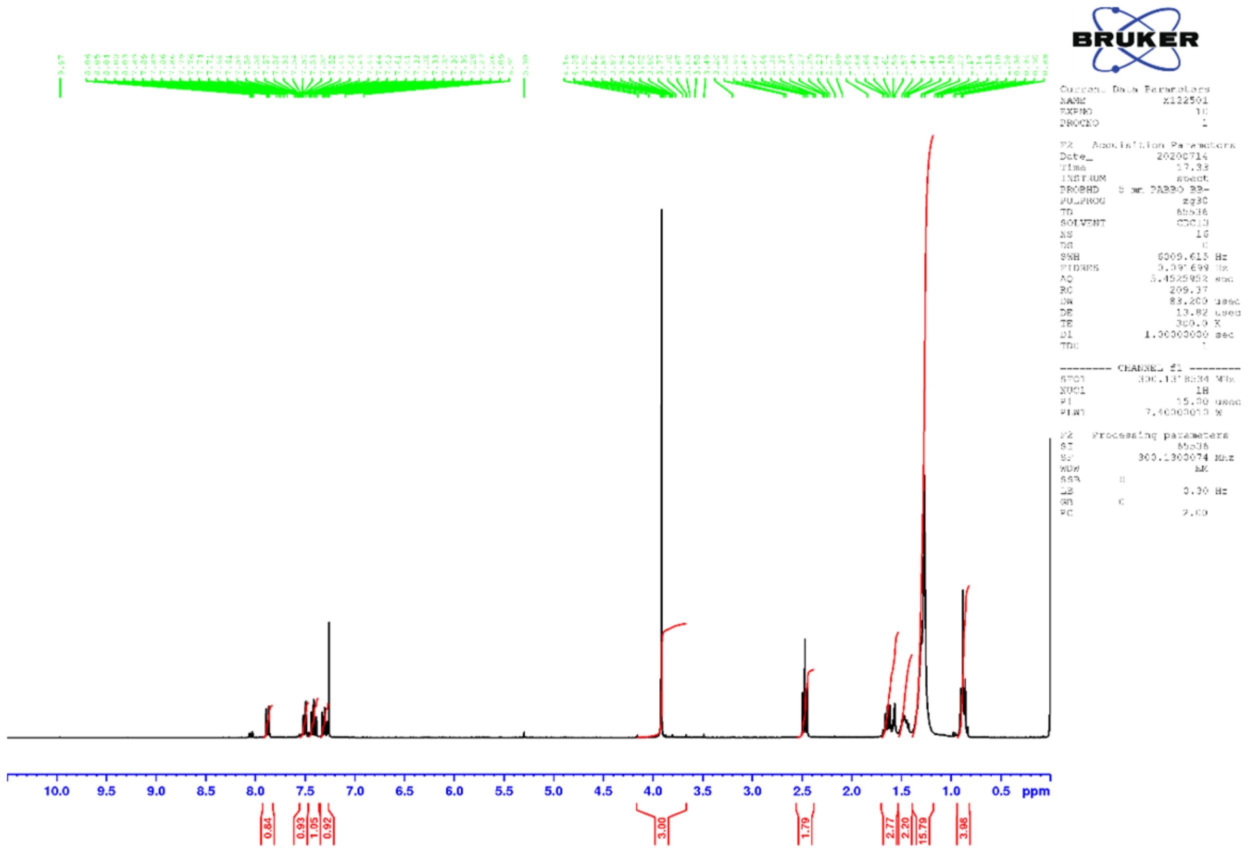

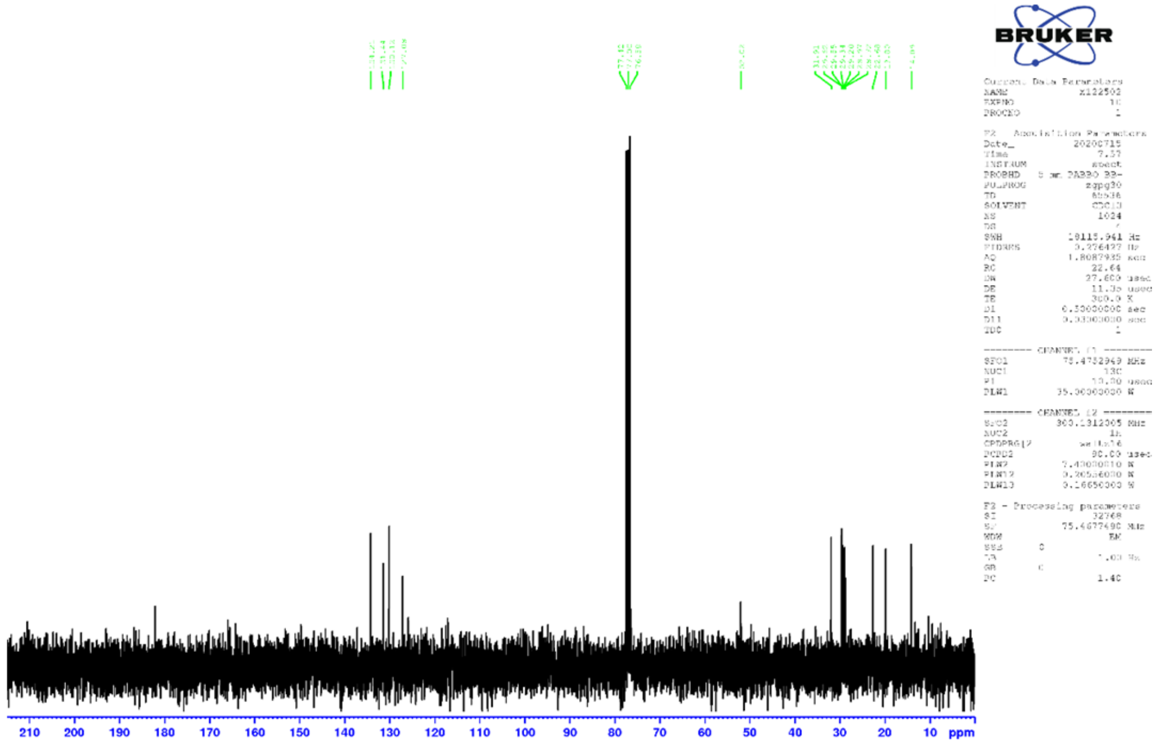

Methyl 2-Tridecylbenzoate (35)

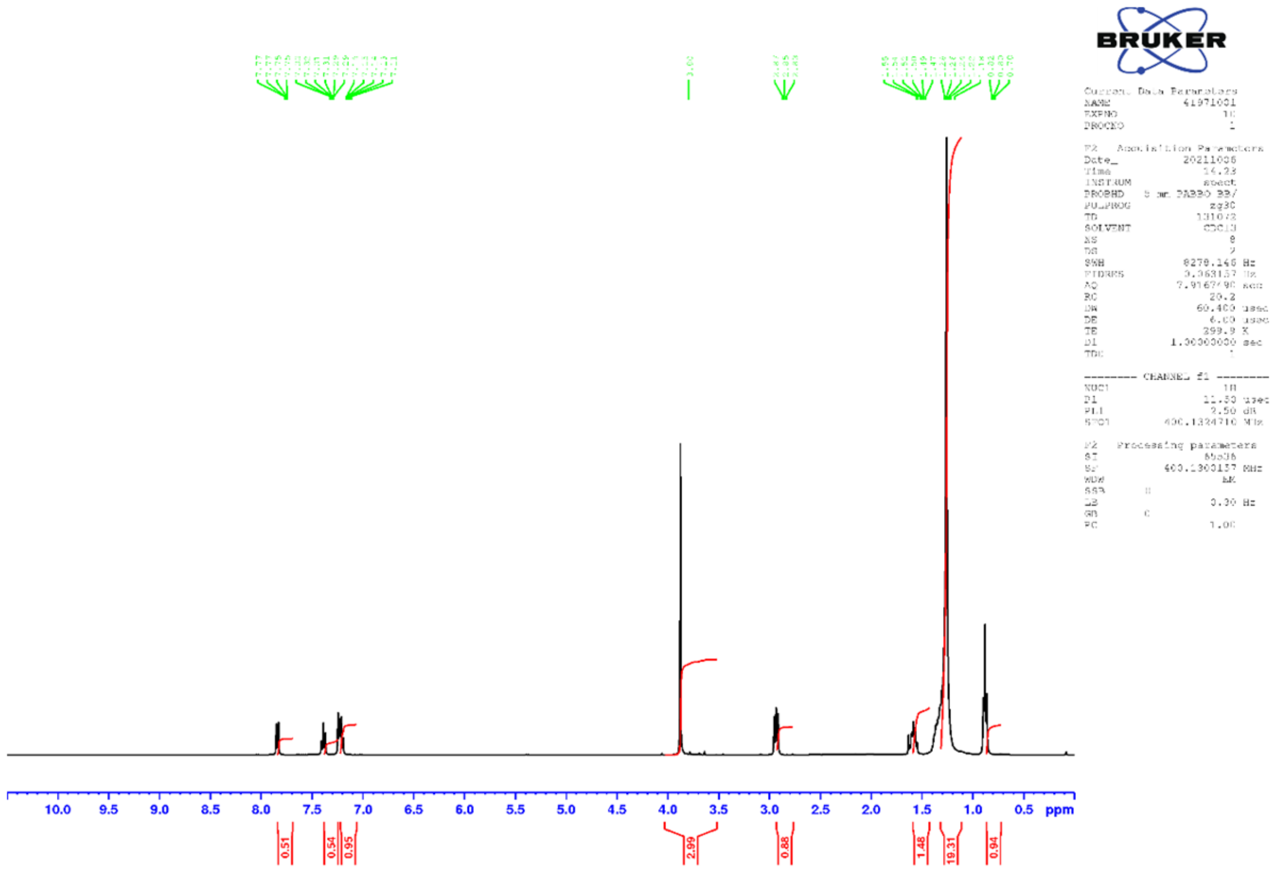

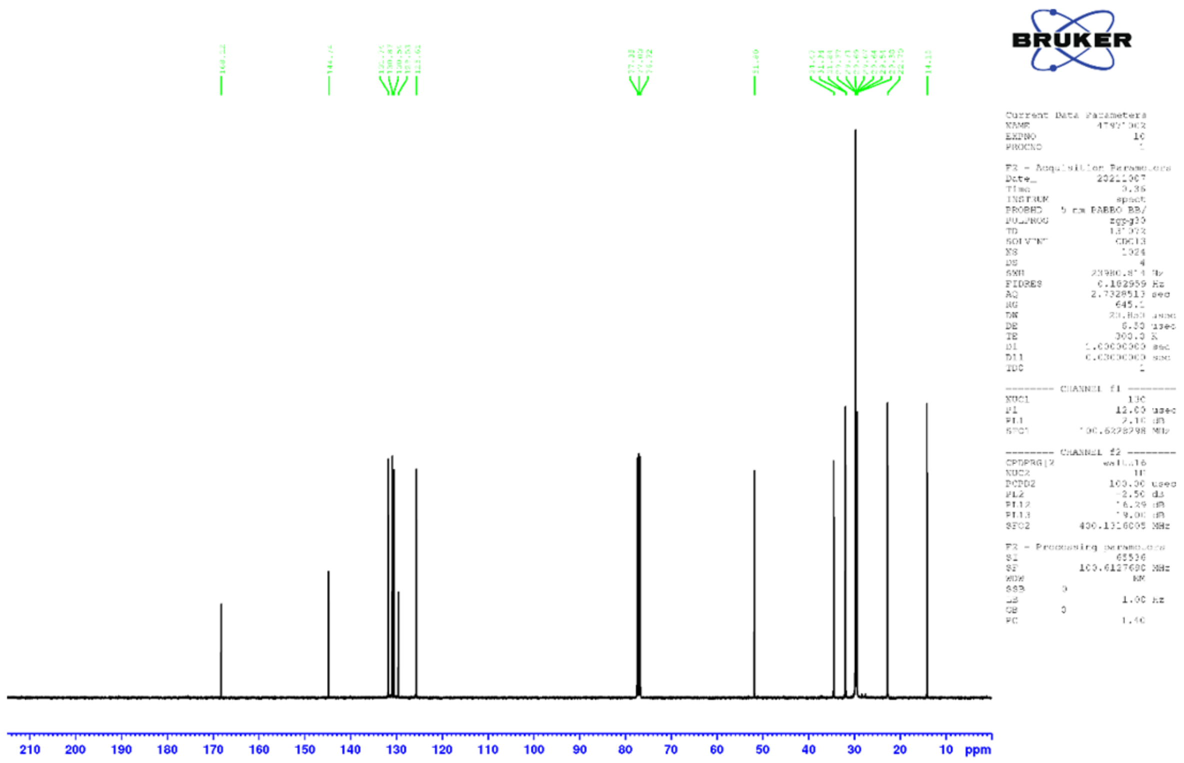

2-Tridecylbenzoic Acid (36)

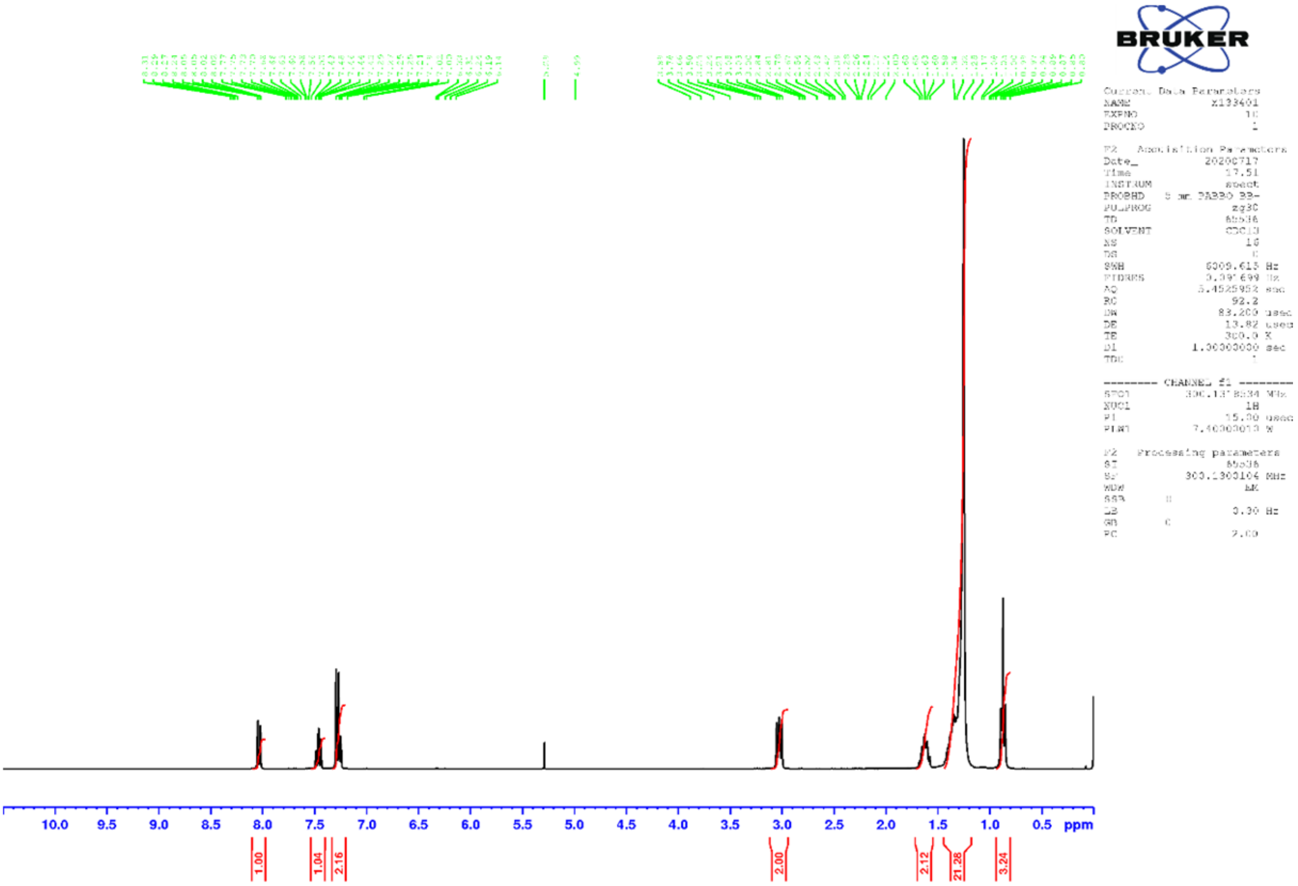

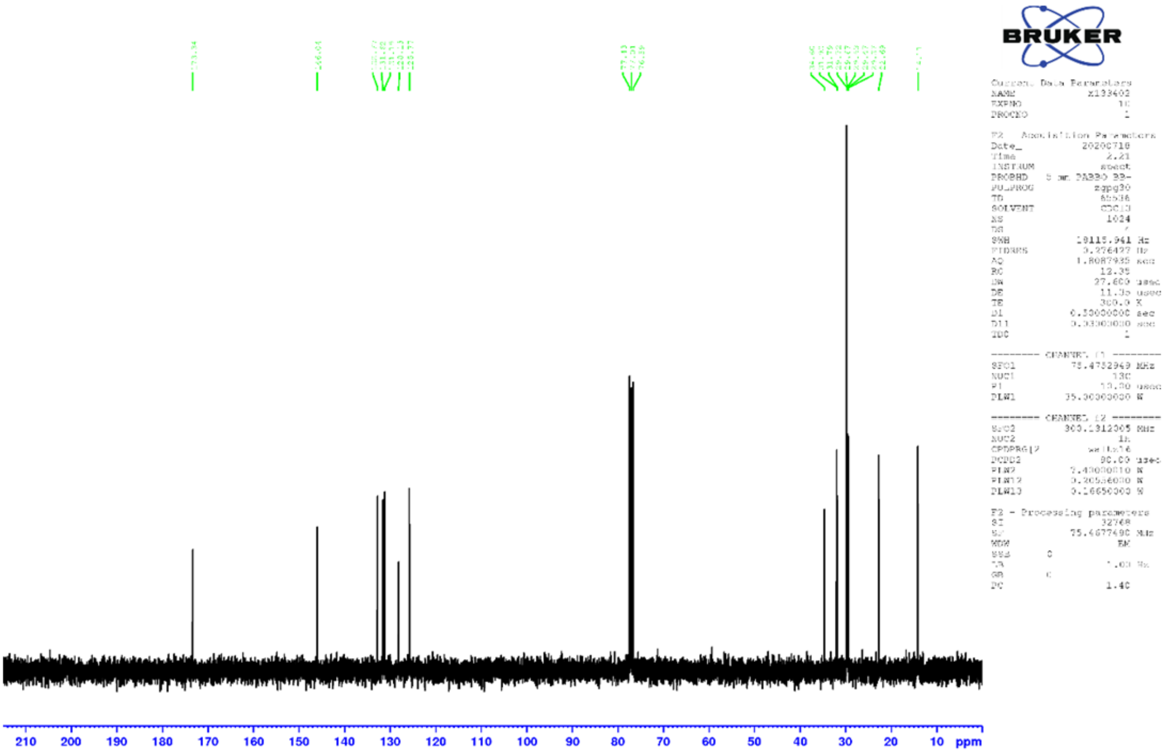

N-(*tert*-Butylsulfonyl)-2-tridecylbenzamide (37)

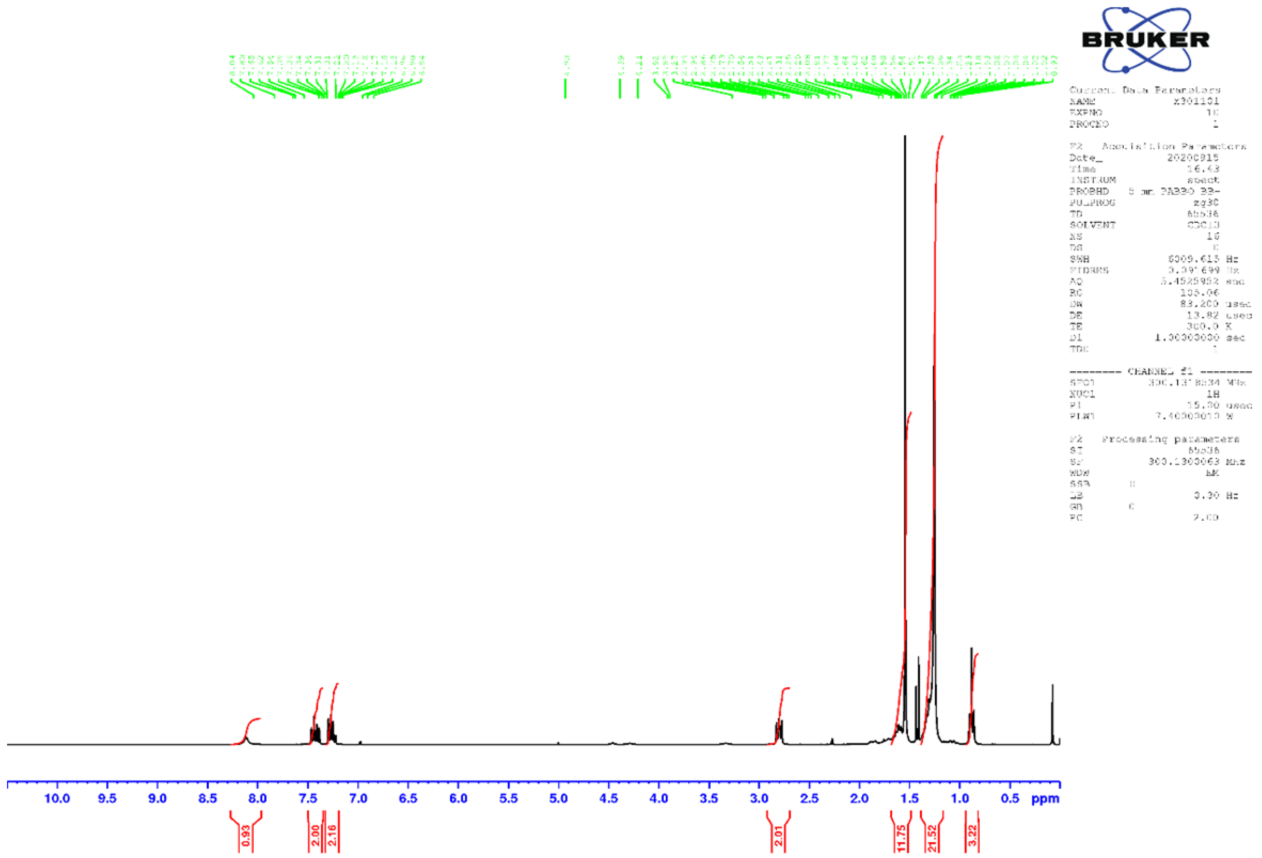

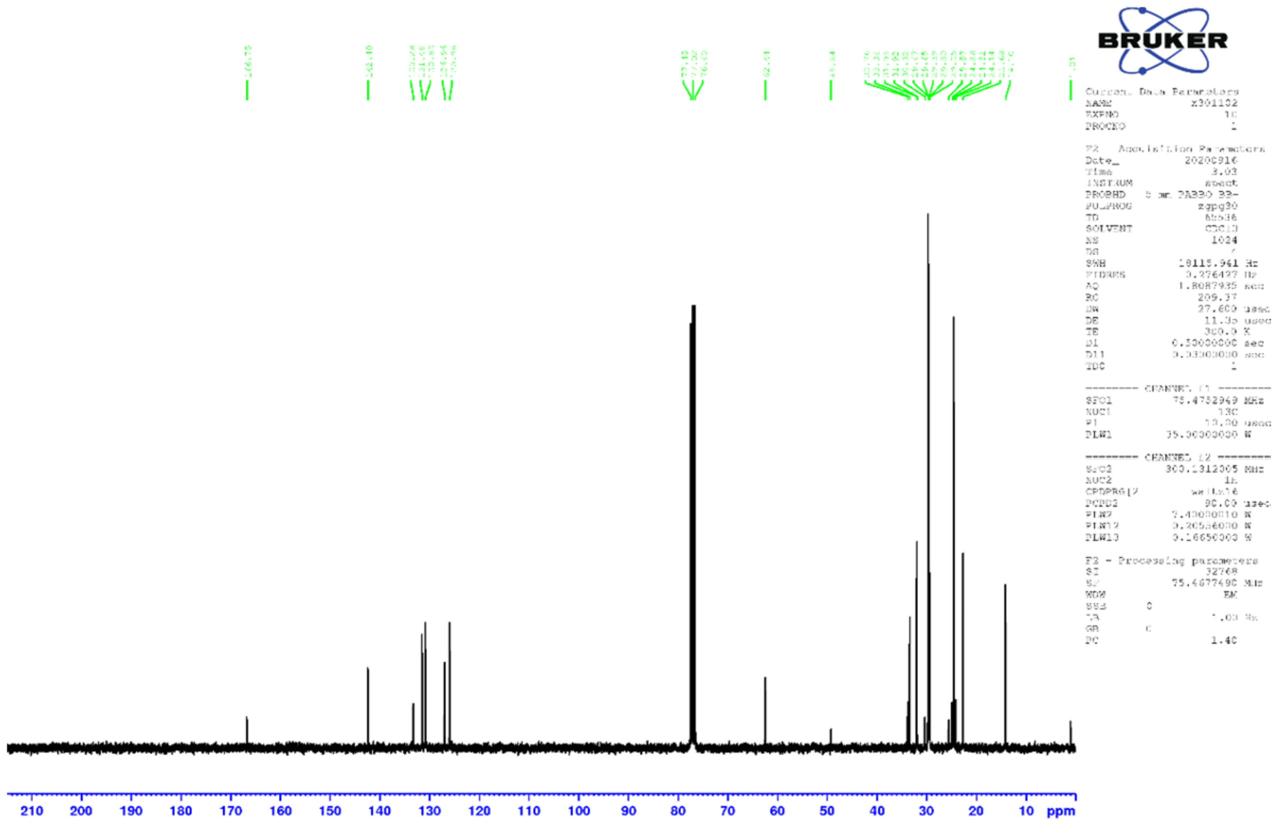

N-((4-Chlorophenyl)sulfonyl)-2-tridecylbenzamide (38)

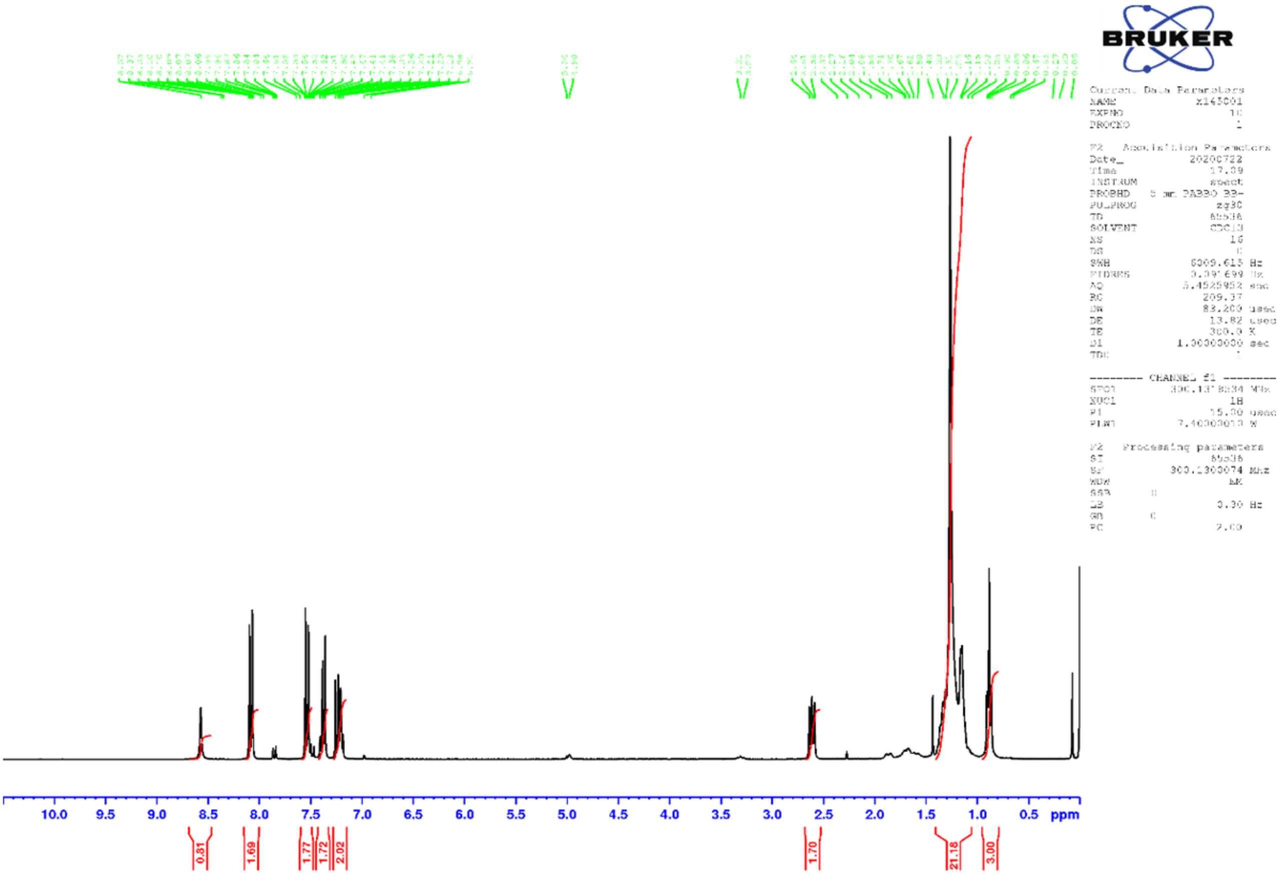

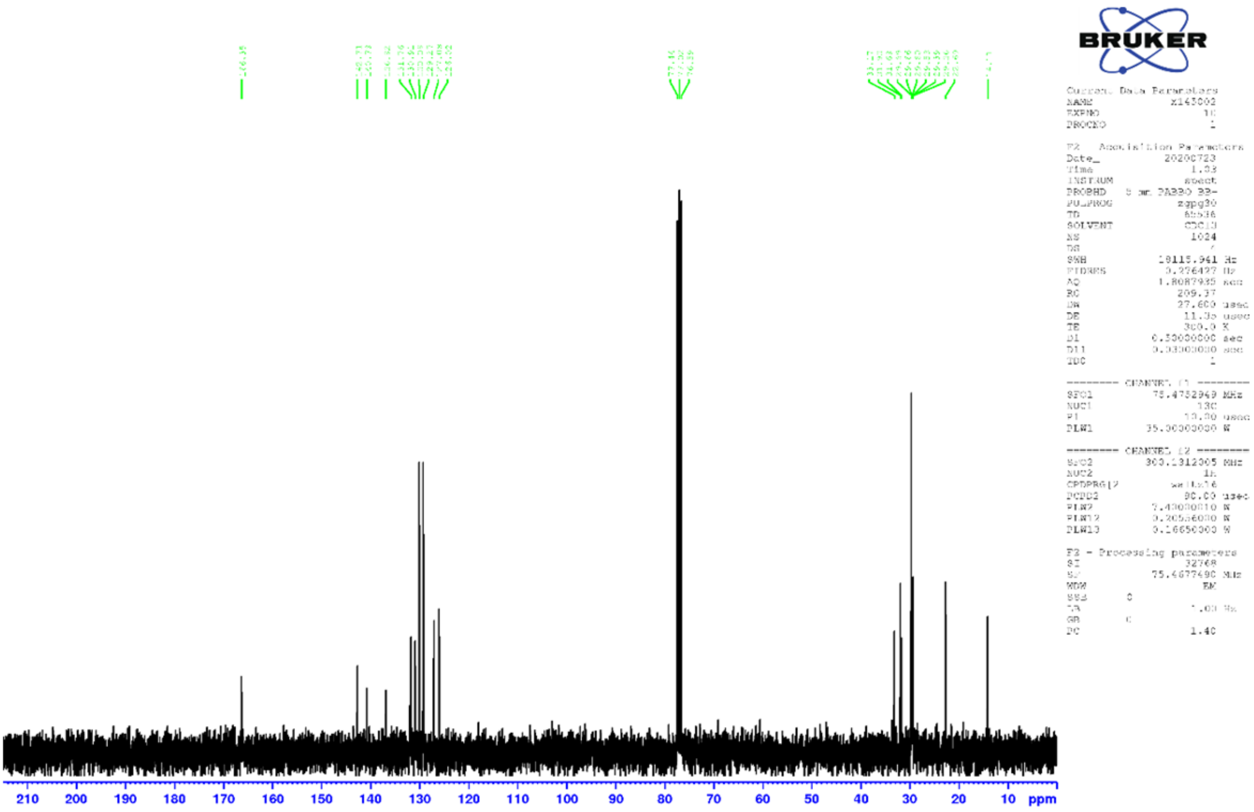

Supplement: Supplementary file 1 [file microorganisms-12-02496-s001.zip › microorganisms-3279161-supplementary.pdf]
